# Decoding the Human Immunoglobulin G-Glycan Repertoire Reveals a Spectrum of Fc-Receptor- and Complement-Mediated-Effector Activities

**DOI:** 10.3389/fimmu.2017.00877

**Published:** 2017-08-02

**Authors:** Gillian Dekkers, Louise Treffers, Rosina Plomp, Arthur E. H. Bentlage, Marcella de Boer, Carolien A. M. Koeleman, Suzanne N. Lissenberg-Thunnissen, Remco Visser, Mieke Brouwer, Juk Yee Mok, Hanke Matlung, Timo K. van den Berg, Wim J. E. van Esch, Taco W. Kuijpers, Diana Wouters, Theo Rispens, Manfred Wuhrer, Gestur Vidarsson

**Affiliations:** ^1^Sanquin Research and Landsteiner Laboratory, Department Experimental Immunohematology, Academic Medical Centre, University of Amsterdam, Amsterdam, Netherlands; ^2^Sanquin Research and Landsteiner Laboratory, Department Blood Cell Research, Academic Medical Centre, University of Amsterdam, Amsterdam, Netherlands; ^3^Center for Proteomics and Metabolomics, Leiden University Medical Center, Leiden, Netherlands; ^4^Sanquin Research and Landsteiner Laboratory, Department Immunopathology, Academic Medical Centre, University of Amsterdam, Amsterdam, Netherlands; ^5^Sanquin Reagents, Amsterdam, Netherlands

**Keywords:** immunoglobulin G glycosylation, Fc gamma receptor, antibody-dependent cellular cytotoxicity, complement, antibody effector functions

## Abstract

Glycosylation of the immunoglobulin G (IgG)-Fc tail is required for binding to Fc-gamma receptors (FcγRs) and complement-component C1q. A variety of IgG1-glycoforms is detected in human sera. Several groups have found global or antigen-specific skewing of IgG glycosylation, for example in autoimmune diseases, viral infections, and alloimmune reactions. The IgG glycoprofiles seem to correlate with disease outcome. Additionally, IgG-glycan composition contributes significantly to Ig-based therapies, as for example IVIg in autoimmune diseases and therapeutic antibodies for cancer treatment. The effect of the different glycan modifications, especially of fucosylation, has been studied before. However, the contribution of the 20 individual IgG glycoforms, in which the combined effect of all 4 modifications, to the IgG function has never been investigated. Here, we combined six glyco-engineering methods to generate all 20 major human IgG1-glycoforms and screened their functional capacity for FcγR and complement activity. Bisection had no effect on FcγR or C1q-binding, and sialylation had no- or little effect on FcγR binding. We confirmed that hypo-fucosylation of IgG1 increased binding to FcγRIIIa and FcγRIIIb by ~17-fold, but in addition we showed that this effect could be further increased to ~40-fold for FcγRIIIa upon simultaneous hypo-fucosylation and hyper-galactosylation, resulting in enhanced NK cell-mediated antibody-dependent cellular cytotoxicity. Moreover, elevated galactosylation and sialylation significantly increased (independent of fucosylation) C1q-binding, downstream complement deposition, and cytotoxicity. In conclusion, fucosylation and galactosylation are primary mediators of functional changes in IgG for FcγR- and complement-mediated effector functions, respectively, with galactose having an auxiliary role for FcγRIII-mediated functions. This knowledge could be used not only for glycan profiling of clinically important (antigen-specific) IgG but also to optimize therapeutic antibody applications.

## Introduction

The importance of the biological properties of antibodies to specifically engage a target of choice and activate complement and Fc gamma receptors (FcγR) on immune cells (1) is currently more and more recognized in modern medicine. For cancer therapies using tumor targeting antibodies, strong effector functions are preferred (2). Various strategies have been exploited to generate antibodies that are more effective than wild-type human IgG1 isotype (3). These include fusions with toxic molecules and incorporations of mutations that enhance affinities to FcγR. Possible drawback of such modifications is the introduction of foreign immunogenic epitopes that can result in anti-drug antibodies that may neutralize the drug. This can be circumvented by using non-immunogenic natural variations, found in all individuals. The prototypic variation of this kind are glyco-engineered IgG1 antibodies without fucose with elevated FcγRIIIa affinities (4, 5), which have already found its way to therapeutic antibodies on the market (6).

This fucose residue is part of a conserved glycan on asparagine 297 in the Fc domain of immunoglobulin G (IgG). This glycan is important for the quaternary structure of the Fc part, since its removal abrogates binding of FcγR and C1q and hence the antibody’s effector functions (7–9). In addition to affecting the Fc structure and thereby recognition by these effector molecules, the Fc-glycan also affects binding to FcγRIIIa and FcγRIIIb through a glycan–glycan interaction (10, 11). This is because of a unique glycan found in human FcγRIIIa and FcγRIIIb at position 162 that interacts directly with the Fc-glycan within the IgG-Fc cavity (11).

The N297 glycan is a bi-antennary complex glycan composed of a constant part with a core consisting of *N*-acetylglycosamines and mannoses and can be found in human serum with variable levels of core fucose, bisecting *N*-acetylglycosamine, galactose, and terminal sialic acids (12). The N-glycans of total serum/plasma IgG consists on average of high fucose levels (95%), low bisection (15%), intermediate levels of galactose (45%), and low sialic acid (10%) (12). The variable assembly of the glycans amounts to at least 20 different glycoforms (a term used here to describe one unique glycan combination) for each IgG subclass being found in serum, with ~8 of them accounting for 90% of the total abundance (12). The composition of total IgG glycosylation can change upon certain settings, where galactosylation and sialylation increase with pregnancy (12, 13). Changes in total IgG are also observed in various clinical settings, with a low level of galactosylation and sialylation associated both with increasing age and autoimmune diseases (12–15).

We and others have shown that IgG-Fc glycosylation changes of antigen-specific IgG can occur that correlate with disease outcome (16–20). This includes both auto- and alloimmune disorders, including fetal neonatal immune thrombocytopenia (FNAIT), immune thrombocytopenia, and hemolytic disease of the fetus and newborn (HDFN) (16–18, 21, 22). In particular, we have found that immune responses against red blood cell (RBC) and platelets, either transfused or during pregnancy, can be characterized with extremely low fucose (down to 10%), high galactose (up to 80%), and elevated sialylation levels (~35%). Notably, lowered Fc-fucosylation (17, 21), but also elevated Fc-galactosylation (18), seemed to correlate with elevated blood cell destruction, severity of anemia or bleeding for RBCs and platelets, respectively. Whereas the increased pathogenicity associated with lowered fucosylation could be explained by the resulting elevated FcγRIIIa and/or FcγRIIIb activity (17, 23), the functional reasons—if any—behind the association with elevated galactosylation remained enigmatic.

The effect of Fc-bisection and -sialylation on human FcγR binding, if any, has been studied in even less detail, although binding to the human FcγRIIIa does not seem to be affected by sialylation (24). Whether these glycan changes influence binding to C1q, and subsequent complement activation, has not been studied in detail (25, 26). A drawback of all these studies is that the impact of the glycan changes was studied changing only individual end groups, without investigating the possibility that the context of the other glycan changes may have an effect on the antibody effector functions.

The complexity of the glycan-assembly makes investigation into their biological relevance extremely difficult. Previous attempts have generated a handful of defined glycoforms and tested binding to part of the FcγR-repertoire, but a systematic analysis for all possible glycan changes and effector mediators, FcγRs and complement, has never been achieved (24, 26–30). This information could provide the insight in working mechanisms of IgG-based treatments and allow meaningful clinical evaluation of the activity of potentially pathological antibodies such as in FNAIT and HDFN. We have, therefore, developed a set of glyco-engineering tools which specifically alter one of the N-glycan end groups (31) and in the present study we combined these tool to create 20 different natural glycoforms to systematically investigate them with regard to FcγR binding, antibody-dependent cellular cytotoxicity (ADCC), complement binding, and activation.

## Materials and Methods

### Human Samples

Peripheral blood from anonymous, healthy volunteers was obtained with informed, written consent in accordance with Dutch regulations. This study was approved by the Sanquin Ethical Advisory Board in accordance with the Declaration of Helsinki.

Heparinized blood samples were used for isolation of peripheral blood mononuclear cells (PBMCs) or RBCs. NK cell isolation was only performed with blood from well-genotyped donors who do not express FcγRIIc (32) to exclude any possible effects of this receptor. Serum was obtained by allowing blood without anticoagulants to coagulate for 1 h at room temperature (RT) and collecting the supernatant after centrifugation at 950 × *g* for 10 min. Serum of three different volunteers was combined to create a serum pool.

### Strains and Reagents

*Escherichia coli* strain DH5α was used for recombinant DNA work. Restriction endonucleases, DNA modification enzymes were obtained from Thermo Fisher Scientific (Waltham, MA, USA). Oligonucleotides were obtained from Geneart (Thermo Fisher Scientific) or Integrated DNA Technologies (Coralville, IA, USA).

### IgG1 Expression Vector Constructs

Variable (V) genes for anti-human RhD (anti-D clone 19A10) heavy and light chain were sequenced from a single human B cell from a hyper immunized donor (33). A single-gene vector containing anti-D or anti-TNP IgG1 heavy- and kappa light-chain-encoding sequences were cloned as described previously by Kruijsen et al. (34) into a pEE14.4 (Lonza, Basel, Switzerland) expression vector. For both anti-TNP and anti-D IgG, a single expression vector was generated. In brief, the codon-optimized V gene for both heavy and light chain, including 5′-HindIII and 3′-NheI or 5′-HindIII and 3′-XhoI restriction sites respectively, Kozak sequence, and HAVT20-leader sequence, were designed and ordered from Geneart (Thermo Fisher Scientific). The HindIII-NheI or HindIII-XhoI fragments for the codon-optimized heavy or light chain were ligated into γ or κ constant region flanking 3′-EcoRI restriction site, respectively. The HindIII–EcoRI fragment for the codon-optimized light chain was ligated into pEE14.4 (Lonza), and the HindIII–EcoRI fragment for the heavy chain was ligated into pEE6.4 (Lonza). A single-gene vector encoding IgG1 was subsequently generated by ligation of the BamHI–NotI fragment from pEE6.4 (including a cytomegalovirus promoter), IgG1 heavy chain, and poly (A) into the light-chain-encoding pEE14.4 vector.

### IgG1 Production and Glyco-Engineering

IgG1 production in human embryonic kidney (HEK) F cells and purification using protein A affinity chromatography was performed as described previously by Kruijssen et al. (34) Glyco-engineering of IgG1 was optimized as described by Dekkers et al. (31) In short, to decrease either fucosylation or galactosylation, 0.4 mM 2-deoxy-2-fluoro-l-fucose (2FF) (Carbosynth, Berkshire, United Kingdom) or 1 mM 2-deoxy-2-fluoro-d-galactose (2FG) (Carbosynth), respectively, was added to the cell suspension 4 h post transfection. To increase bisecting GlcNAc, 1% pEE6.4 + GNTIII encoding mannosyl (beta-1,4-)-glycoprotein beta-1,4-*N*-acetylglucosaminyltransferase (GNTIII) enzyme was co-transfected with 99% IgG1-κ HC + LC vector. To increase galactose, 1% pEE6.4 + B4GALT1 encoding β-1,4-galactosyltransferase 1 (B4GALT1) enzyme was co-transfected with 99% IgG1 vector and 5 mM d-galactose (Sigma Aldrich, Saint Louis, MO, USA) was added to the cell suspension 1 h before transfection. To increase sialylation, the level of galactosylation must also be elevated as sialic acid is the terminal sugar group with galactose residues as substrate. Thus, 1% pEE6.4 + B4GALT1 and 2.5% pEE14.4 + STGALT encoding β-galactoside alpha-2,6-sialyltransferase 1 (ST6GALT) were both co-transfected 96.5% IgG1 vector and 5 mM d-galactose was added to the cell suspension 1 h before transfection. To further increase sialylation, *in vitro* sialylation (ivs) was performed on the purified *in vivo* sialylated IgG created using the previous method. Recombinant human α-2,6-sialyltransferase (Roche, Basel, Switzerland) and cytidine-5′-monophospho-*N*-acetylneuraminic acid (CMP-NANA) (Roche) were incubated at 37°C for 24 h with purified IgG1 with already *in vivo* enhanced galactose and sialic acid (as described above), after incubation samples were re-purified with protein A, as described previously (31, 34).

### Mass Spectrometry Analysis

Immunoglobulin G-Fc glycan composition of produced IgG1 was determined by mass spectrometry as described previously by Dekkers et al. (31) Trypsin-digested glycopeptide samples were analyzed by nanoLC–ESI–QTOF–MS. The separation was performed on an RSLCnano Ultimate 3000 system (Thermofisher, Breda, the Netherlands) with a gradient pump, loading pump and an autosampler. 250 nl of sample was injected and washed on a Dionex Acclaim PepMap100 C18 trap column (5 mm × 300 µm i.d.; Thermofisher) for 1 min with 0.1% TFA at a flow rate of 25 µl/min. The sample was then separated on an Ascentis Express C18 nanoLC analytic column (50 mm × 75 µm i.d.; 2.7-µm fused core particles; Supelco, Bellefonte, PA) with a flow rate of 0.9 µl/min using linear gradient as described in Ref (30). The resulting co-elution of the different glycoforms of the IgG1-Fc glycosylation site warrants fair comparison by ensuring identical ionization conditions for the various glycopeptide species. The LC was coupled to the MS detector *via* a CaptiveSpray source with a NanoBooster (Bruker Daltonics, Bremen, Germany). The latter enriched the N_2_ flow (3 l/min) with CH_3_CN (pressure 0.2 bar), resulting in increased sensitivity. The samples were ionized in positive ion mode at 1,100 V. The Maxis Impact quadrupole-TOF–MS (micrOTOF-Q, Bruker Daltonics) was used as detector. MS1 spectra were collected at a frequency of 1 Hz with a scan range of *m/z* 550–1,800. The mass spectrometric data were calibrated internally in DataAnalysis 4.0 (Bruker Daltonics) using a list of known IgG glycopeptide masses. MSConvert (Proteowizard 3.0) (35) was used to convert the data files to mzXML format, and an in-house alignment tool (36) was used to align the retention times of the data files. The highest intensity of selected peaks (within an *m/z* window of ±0.2 and within a time window of ±15 s surrounding the retention time) was extracted using the in-house developed 3D Max Xtractor software tool. If above a signal:background ratio of 3, the background-subtracted area of the first three isotopic peaks of each glycopeptide in both 2+, 3+, and 4+ charge state were summed, and this summed value was then divided by the total summed value of all IgG1 glycopeptides to arrive at a percentage for each glycopeptide. From these percentages, we calculated several derived traits using the following formulas: fucosylation (H3N3F1 + H4N3F1 + H5N3F1 + H6N3F1 + G0F + G1F + G2F + H6N4F1 + G0FN + G1FN + G2FN + H6N5F1 + H4N3F1S1 + H5N3F1S1 + H6N3F1S1 + G1FS + G2FS + H6N4F1S1 + G2FS2 + G1FNS + G2FNS + H6N5F1S1 + G2FNS2), bisection (H6N4F1 + G0FN + G1FN + G2FN + H6N5F1 + H6N4F1S1 + G1FNS + G2FNS + H6N5F1S1 + G2FNS2 + H6N4 + G0N + G1N + G2N + H6N5 + H6N4S1 + G1NS + G2NS + H6N5S1 + G2NS2), galactosylation [(H4N3F1 + H5N3F1 + G1F + H6N4F1 + G1FN + H6N5F1 + H4N3F1S1 + H5N3F1S1 + H6N3F1S1 + G1FS + H6N4F1S1 + G1FNS + H6N5F1S1 + H4N3 + H5N3 + H6N3 + G1 + H6N4 + G1N + H6N5 + H4N3S1 + H5N3S1 + H6N3S1 + G1S + H6N4S1 + G1NS + H6N5S1) * 0.5 + G2F + G2FN + G2FS + G2FS2 + G2FNS + G2FNS2 + G2 + G2N + G2S + G2S2 + G2NS + G2NS2], sialylation [(H4N3F1S1 + H5N3F1S1 + H6N3F1S1 + G1FS + G2FS + H6N4F1S1 + G1FNS + G2FNS + H6N5F1S1 + H4N3S1 + H5N3S1 + H6N3S1 + G1S + G2S + H6N4S1 + G1NS + G2NS + H6N5S1) * 0.5 + G2FS2 + G2FNS2 + G2S2 + G2NS2], hybrid-types (H5N3F1 + H6N3F1 + H6N4F1 + H6N5F1 + H5N3F1S1 + H6N3F1S1 + H6N4F1S1 + H6N5F1S1 + H5N3 + H6N3 + H6N4 + H6N5 + H5N3S1 + H6N3S1 + H6N4S1 + H6N5S1), and high-mannose (H5N2 + H6N2 + H7N2 + H8N2 + H9N2). For some of the minor hybrid-type glycans, it could not be determined conclusively whether a galactose or a bisecting *N*-acetylglucosamine was present, so an educated guess was made based on structural knowledge (for instance, since the hybrid glycan H6N4F1 is elevated in GNTIII-co-transfected HEK cell-derived IgG samples, it is likely to be a bisected species rather than triantennary).

### High-Performance Liquid Chromatography (HPLC)

Protein A purified IgG was analyzed for monomeric and dimeric IgG on a Superdex 200 10/300 gel filtration column (30 cm, 24 ml, 17-15175-01, GE Healthcare, Little Chalfont, United Kingdom) connected to an Äkta explorer (GE Healthcare) HPLC system at RT with a flow rate of 0.5 ml/min and PBS as running buffer. Elution profiles were obtained by recording the absorbance at 215 nm.

### Human FcγR Constructs

Human FcγR constructs [FcγRIa (HIS tag), FcγRIIa (131His, Biotinylated, and 131Arg, Biotinylated), FcγRIIb (Biotinylated), FcγRIIIa (158Phe, Biotinylated, and 158Val, Biotinylated) and FcγRIIIb (NA2, HIS tag)] for surface plasmon resonance (SPR) analysis were obtained from Sino biological (Beijing, China). To further include all human FcγRs, a fusion Fc–FcγR construct composed of the extracellular domain of the FcγRIIIb in both allotypes followed by a Fc domain was created. To create the fusion Fc–FcγRIIIb constructs the amino acid code of the extracellular domain of either FcγRIIIb of NA1 allotype or FcγRIIIb NA2 allotype (37) (NCBI reference sequence NP_000561.3), and IgG2 Fc domain, composed of a human IgA1a hinge, human IgG2 Fc CH2 and CH3 domains including mutations deleting the Fc-glycan (N297A) and introducing a C-terminal biotinylation tag (BirA) were reverse translated and codon optimized at Geneart. DNA was ordered (Integrated DNA technologies, Coralville, IA, USA) and cloned into pcDNA3.1 (Invitrogen, Carlsbad, CA, USA) expression vector using flanking HindIII and EcoRV restriction sites. A model of the construct and sequences are displayed in Figures S7A,B in Supplementary Material. The construct was produced and purified as described previously (31). After purification the protein was site-specifically biotinylated on the BirA tag using BirA enzyme as described by Rodenko et al. (38). For biotinylation of 1 µM FcγR protein 0.00657 µM BirA ligase was used. After biotinylation overnight at 25°C, the FcγR sample was buffer-exchanged and subsequently concentrated in PBS pH 7.4 using Amicon Ultra centrifugal filter units (MWCO 30 kDa) (Merck, Millipore, Darmstadt, Germany). The quality of the Fc-Fusion receptors was confirmed by comparing the binding of normally glycosylated IgG1 to the acquired his-tagged receptor (Sino-biological) and in-house made Fc-Fusion of the same allotype (NA2) (Figures S7C,D in Supplementary Material).

### Surface Plasmon Resonance

Surface plasmon resonance measurement were performed as described by Dekkers et al. (39). All biotinylated FcγR were spotted using a Continuous Flow Microspotter (Wasatch Microfluidics, Salt Lake City, UT, USA) onto a single SensEye G-streptavidin sensor (Ssens, Enschede, Netherlands) allowing for binding affinity measurements of each antibody to all FcγR simultaneously on the IBIS MX96 (IBIS Technologies, Enschede, Netherlands) as described by de Lau et al. (40). The biotinylated FcγRs were spotted in threefold dilutions, ranging from 100 to 3 nM for FcγRIIb and fusion FcγRIIIb-IgG2-Fc. All the other FcγRs were spotted in threefold dilutions, ranging from 30 to 1 nM in PBS 0.0075% Tween-80 (Amresco), pH 7.4. The IgGs were then injected over the IBIS at 1.5 dilution series starting at 5.9 nM until 506.25 nM or 0.9 nM until 2,000 nM, when necessary, in PBS in 0.075% Tween-80. For FcγRI affinity and FcγRIIIb control measurements, his-tagged FcγRI or FcγRIIIb was used. Biotinylated anti-His-tagged antibody (Genscript Piscataway, NJ, USA) was spotted in threefold dilutions, ranging from 30 to 1 nM. Before every IgG injection, 50 nM his-tagged FcγR was injected. The IgGs were then injected over the IBIS at threefold dilution series starting at 0.41 nM until 100 nM for FcγRI and 94 nM until 3,000 nM for FcγRIIIb. Regeneration after every sample was carried out with acid buffer (10 mM Gly–HCl, pH 2.4). Calculation of the dissociation constant (*K*_D_) was done using an equilibrium analysis by linear intrapolation to Rmax = 500 (41). Analysis and calculation of all binding data were carried out with Scrubber software version 2 (Biologic Software, Campbell, ACT, Australia) and Microsoft Office Excel 2013.

### NK Cell-Mediated ADCC

NK cells were isolated from Ficoll-Plaque™-Plus (GE Healthcare) gradient obtained PBMCs by a CD56 magnetic-activated cell separation isolation kit (Miltenyi Biotec, Leiden, The Netherlands), according to manufacturer’s description. D + RBCs were isolated and labeled with radioactive chromium (100 μCi ^51^Cr, PerkinElmer, Waltham, MA, USA) at 10^9^ cells/ml. An amount of 10^5^ erythrocytes were incubated with NK cells for 2 h at 37°C in a 2:1 ratio in Iscove’s modified dulbecco’s medium (IMDM, Gibco, Thermo Fisher Scientific) supplemented with 10% fetal calf serum (FCS, Bodinco, Alkmaar, The Netherlands) and anti-D IgG1-glycoforms at a total volume of 100 µl. To determine 100% lysis, 2.5% saponine (Fluka, Sigma Aldrich) was added to RBC in control wells and spontaneous lysis (sp) was determined by incubation of RBC without NK cells. Supernatants were collected and released ^51^Cr was quantified in a Packard Cobra II Auto-Gamma Counter Model D5005 (PerkinElmer). Percentage cytotoxicity was determined by the following formula: $\text{ADCC}\left(\%\right)=\frac{\text{counts sample}-\text{counts sp}}{\text{counts 100\%}-\text{counts sp}}\times 100$, each value consisted of at least three individual sample wells.

### Complement Deposition ELISA

A 2.4-mM 2,4,6-trinitrobenzenesulfonic acid (TNBS) (Sigma-Aldrich) solution was added to 20 mg human serum albumin (HSA) diluted to 20 mg/ml (Sanquin, Amsterdam, The Netherlands) in 0.2 M NA_2_HPO_4_ (Merck, Millipore) and incubated 30 min at RT. To remove unbound TNBS, the solution was dialyzed (1:2,000) using a dialysis cassette (Thermo Fisher Scientific Slide-A-Lyzer G2 cassette, 10K MWCO) for 1.5 h at RT against PBS and additionally overnight at 4°C to obtain HSA-TNP.

To coat, maxisorp plates (Thermo Scientific, Nunc flat-bottom 96-well plate) were incubated o/n at RT with 20 µg/ml HSA-TNP in PBS. The plates were washed 5× with PBS + 0.1% tween-20 (Sigma-Aldrich) (wash buffer) using an ELISA washer (Biotek, 405 LSRS). All following washing steps were done similarly. The IgG samples were diluted in 100 µl PBS/plx [PBS + 0.1% poloxamer (Sigma-Aldrich, poloxamer 407)] per well and incubated for 1.5 h at RT. The plates were washed and incubated with 100 µl 1:35 serum pool in VB^+/+^/plx {veronalbuffer [3 mM Barbital (Sigma-Aldrich), 1.8 mM Sodium-Barbital (Sigma Aldrich), 0.146 M NaCl (Fagron, Capelle aan den Ijssel, The Netherlands), pH 7.4] + 10 mM CaCl_2_ (Merck) + 2 mM MgCl_2_ (Merck) + 0.1% poloxamer} for 1 h at RT. When C1q was blocked, 10 min prior to addition of serum to the ELISA plate, anti-C1q-85 blocking antibody (42) was added to the VB^+/+^/plx + 1:35 serum solution in a 1:2 molar ratio of C1q:anti-C1q-85 with final concentration of 8.57 µg/ml anti-C1q-85. The plates were washed and 100 µl with either 2 µg/ml biotinylated anti-C1q-2 (42), 0.5 µg/ml biotinylated anti-C4-10 (43), 0.6 µg/ml biotinylated anti-C3-19 (44), or 1 µg/ml HRP labeled anti-human IgG (Sanquin, Peliclass) in PBS/plx was added to respectively detect C1q, C4b, C3b, or IgG deposition and incubated for 1 h at RT. The plates were washed, C1q, C4b, and C3 plates were incubated with 100 µl 0.2 µg/ml strep-poly HRP (Sanquin, Peliclass) (C1q) or 0.25 µg/ml strep-HRP (Sigma-Aldrich) (C4b and C3b) in PBS/plx for 1 h at RT. The plates were washed and developed for 5–10 min using 100 µl TMB mix composed of 0.11 M NaAc (pH 5.5) (Merck), 0.1 mg/ml 3,3′,5,5′-Tetramethylbenzidine (Merck) and 0.003% H_2_O_2_ (Merck) and the reaction was stopped with the addition of 100 µl 2 M H_2_SO_4_ (Merck). The optical density (OD) was measured at A450 nm using a plate reader (Biotek, Synergy 2, Winooski, VT, USA).

The results were analyzed with a parallel line assay in Microsoft Office Excel (45). We assessed the potency of the glycoforms relative to a standard, an independently titrated unmodified IgG1; these values were expressed as percentages relative to the unmodified glycoform.

### Complement-Mediated Lysis

Fifty microliters of washed, packed, D^+^ RBCs obtained from heparinized blood were mixed with 350 µl 0.313 mM TNBS in 0.15 M Na_2_HPO_4_, pH 8.8 and incubated for 10 min at RT. TNPylated RBCs were centrifuged for 2 min at 350 × *g* and washed two times with PBS. RBC were resuspended into VBG^+/+^ [VB^+/+^ + 0.05% w/v gelatin (Sigma-Aldrich)]. Anti-TNP IgG1 was serially diluted in VBG^−/−^ (3 mM Barbital, 1.8 mM Sodium-Barbital, 0.146 M NaCl, pH 7.4, 0.05% w/v gelatin). In round bottom plates to a final volume of 100 µl we combined the diluted IgG1, 10% serum, ~4.5 × 10^6^ RBC, and a glass bead (2 mm, Merck) to ensure mixing of the solution during incubation (1:1 final ratio VBG^−/−^:VBG^+/+^). This amount of RBC was taken to ensure the 100% absorbance between 1.8 and 2.2 delta (Δ) A412–A690 nm. The plates were incubated for 90 min at 37°C while shaking at 150 rpm (Orbital incubator S150, 16 mm shaking diameter). After incubation, 1.25% w/v saponine was supplemented to the 100% control wells, 100 µl VBG^−/−^ was added to all wells and the plates were centrifuged for 2 min at 350 × *g*. Subsequently, 150 µl of supernatant was transferred into a separate plate and OD was measured at Δ A412–A690 nm using a plate reader. The percentage of lysed cells was calculated as follows: $\text{Lysis}\left(\%\right)=\frac{\text{OD sample-OD spontaneous}}{\text{OD 100-OD spontaneous}}\times 100$. In GraphPad Prism, we calculated the half maximal effective concentration (EC_50_) for each replicate of the different glycoforms using a non-linear fit for normalized response with a variable slope and combined these to an average EC_50_.

### Statistical Analysis

Statistical analyses were performed using GraphPad Prism version 6.00 for Windows (GraphPad Software, La Jolla, CA, USA). The level of significance was set at *p* < 0.05 using two-tailed tests.

## Results

### Recapitulation of All 20 Major Different Glycoforms Found in Human Plasma

**Figure 1:**
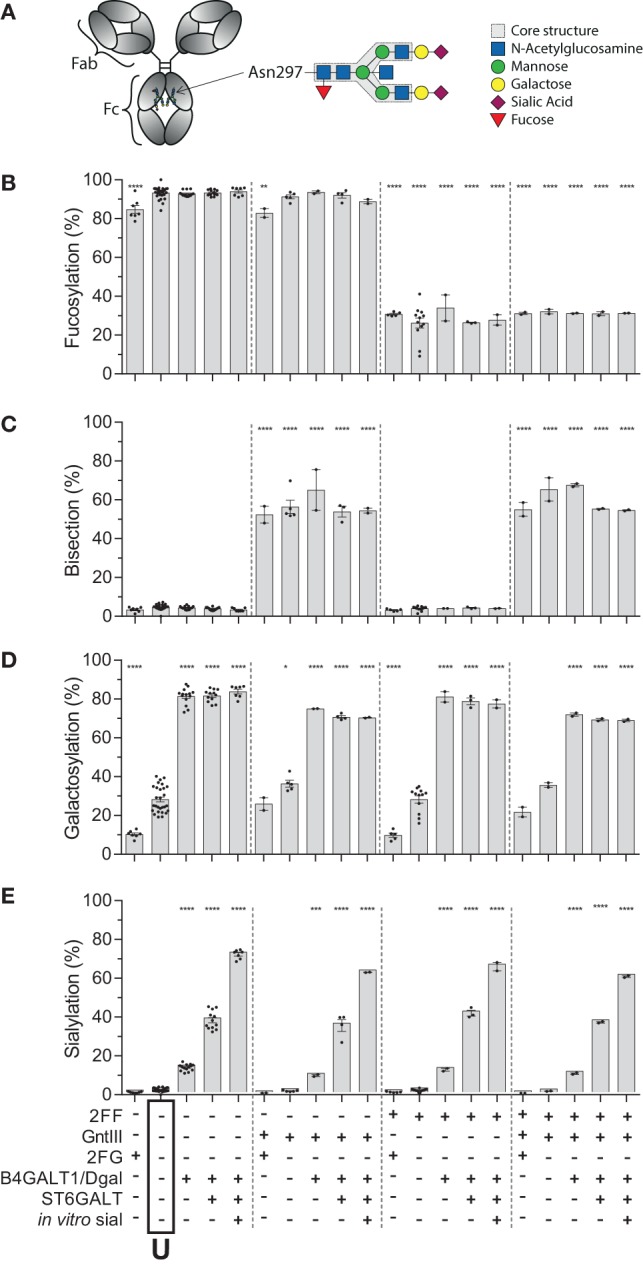
Recapitulation of 20 different immunoglobulin G (IgG) glycoforms by glyco-engineering. **(A)** Model of IgG with glycan at position N297 in the Fc domain and composition of the glycan. **(B–E)** Degree of derived glycan traits as reached by the different glyco-engineering tools: 2FF, 0.4 mM 2-deoxy-fluoro-l-fucose; GntIII, 1% GntIII co-transfection; 2FG, 1 mM 2-deoxy-fluoro-d-galactose; B4galT1/Dgal, 1% B4GALT1 co-transfection and 5 mM d-galactose; ST6GALT, 2.5% ST6GALT co-transfection, *in vitro* sial, treatment of IgG with recombinant ST6GALT and CMP-NANA substrate. The data represent the mean and SEM of at least two combined independent experiments; *, **, ***, and **** denote a statistical significance of *p* ≤ 0.05, *p* ≤ 0.01, *p* ≤ 0.001, and *p* ≤ 0.0001, respectively, as tested by one-way ANOVA against unmodified IgG1, using Dunnett’s multiple comparisons test. U: unmodified glycoform.

Human IgG1, produced in HEK cells, shows complex-type bi-antennary glycans similar to IgG from normal human plasma (Figure 1A) (31, 46). More specifically, without any modification (“Unmodified,” box labeled “U” in the *x*-axis legend, Figures 1B–E) HEK-derived IgG1 N-glycans feature high fucosylation, low bisection, intermediate-level galactosylation, and low sialylation (Figures 1B–E). We previously developed six glyco-engineering tools which can be implemented upon protein production, as we recently described (31). These were aimed to decrease fucosylation, increase bisection, decrease or increase galactosylation, or increase sialylation. In the present study, these tools were combined and used in all possible combinations during the transient transfection in HEK cells, which resulted in the anticipated glycosylation changes and allowed us to produce the 20 major glycoforms present in human serum. Only minor unanticipated effects (Figures 1B–E), were observed. A slight increase in galactosylation upon overexpression of beta 1,4-*N*-acetylglucosaminyltransferase III (GntIII) to increase bisection (e.g., 28 to 36% upon GntIII expression)—but this was only observed in samples with low starting-levels of galactosylation (Figures 1C,D). Some of the tools caused a minor increase (<21%) in high-mannose or hybrid glycan species (Figure S1 and Table S1 in Supplementary Material) (31). Using the glyco-engineering tools the most extreme levels were reached for fucose and galactose (Figures 1B,D), bisection was increased up to 60%, and sialylation never reached over half of what was possible by the underlying galactose (~40%). The level of sialylation was, therefore, further increased using *in vitro* sialylation as described before (up to ~70%) (Figure 1E; Table 1) (31, 47, 48). All in all, this resulted in 20 combinations and markedly different glycoforms. All 20 glycoforms were produced as two panels of IgG1, specific for the RhD (anti-D) antigen or 2,4,6-trinitrophenyl hapten (anti-TNP) (34), with both panels showing highly comparable glycosylation patterns depending of the glyco-engineering tools applied (Table 1; Table S1 in Supplementary Material). To avoid any possible confounding effects of Fab glycosylation on IgG function, we used anti-D and anti-TNP with variable domains sequences devoid of N-linked glycosylation sites.

**Table 1:** Comprehensive list of glycopeptide degrees of complex glycans found in the glyco-engineered IgG1 batches of anti-D and anti-TNP specificity.

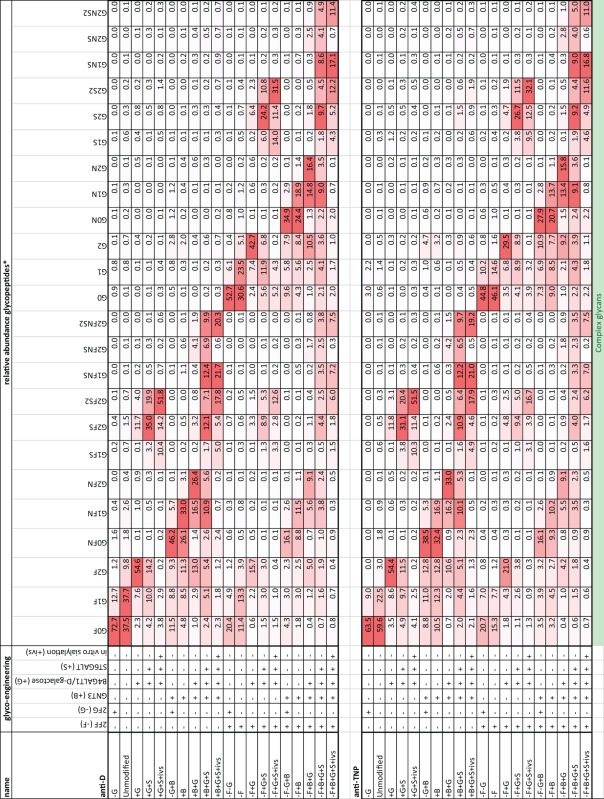

*2FF, 0.4 mM 2-deoxy-2-fluoro-l-fucose; 2FG, 1 mM 2-deoxy-2-fluoro-d-galactose; GNT3, co-transfection of 1% GNT3 vector; B4GALT1/d-galactose, co-transfection of 1% B4GALT1 vector and addition of 5 mM d-galactose; ST6GALT, co-transfection of 2.5% ST6GALT vector; *in vitro* sialylation, treatment of sample with recombinant ST6GALT and CMP-NANA; G, number of galactoses; F, presence of a core fucose; N, presence of a bisecting N-acetylglucosamine; S, number of *N*-acetylneuraminic (sialic) acids*.

*These resulted in significantly different derived glycosylation traits (fucosylation, bisection, galactosylation, sialylation, high-mannose, hybrid-type), which are calculated from the relative abundances of individual *N*-glycans*.

*The shading of cells indicates, for each glycoform the lowest to highest abundance of glycopeptides, respectively, from light to dark*.

*^a^For complex immunoglobulin G glycans, we use a nomenclature which assumes structural knowledge of the glycans based on literature*.

### Binding of IgG Glycome to Human FcγR

**Figure 2:**
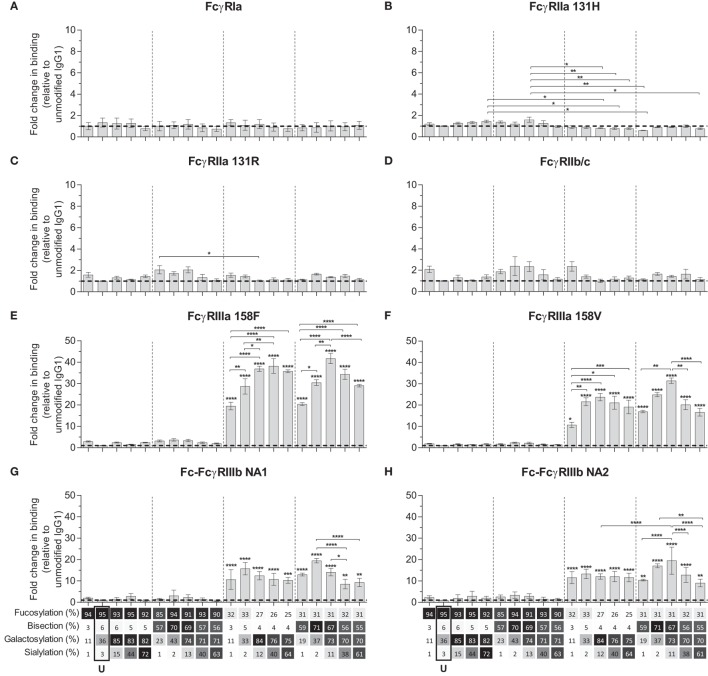
Binding of immunoglobulin G (IgG) glycoforms to human FcγR. Binding of IgG glycoforms to the human FcγR family as determined by surface plasmon resonance, displayed as relative binding compared to unmodified IgG1 (U), **(A)** FcγRI, **(B)** FcγRIIa 131H, **(C)** FcγRIIa 131R, **(D)** FcγRIIb/c, **(E)** FcγRIIIa V158, **(F)** FcγRIIIa F158, **(G)** FcγRIIIb NA1, and **(H)** FcγRIIIb NA2. *x*-Axis legend describes the percentage of each derived glycan trait indicated and by grayscale, from light to dark. The data represent the mean and SEM of at least two combined independent experiments; *, **, ***, and **** (above each column as tested against unmodified, or as indicated, for FcγRIIIs comparing each set of five glycoforms defined by the vertical dotted lines, based on fucose and bisection levels) denote a statistical significance of *p* ≤ 0.05, *p* ≤ 0.01, *p* ≤ 0.001, and *p* ≤ 0.0001, respectively, as tested by one-way ANOVA using Tukey’s multiple comparisons test. U: unmodified glycoform.

We next used the IBIS MX96 biosensor system, as described in Dekkers et al. (39), capable of analyzing the binding of up to 48 different receptor ligand interactions in parallel by SPR, to probe the affinity of all IgG1-glycoforms to all human FcγRs and their allotypes affecting IgG binding (Table S2 in Supplementary Material) (49). The antibodies used for these experiments (anti-D) showed no signs of dimers or multimers (Figure S2 in Supplementary Material). The binding affinities of unmodified IgG1 to the different receptors resembled those reported earlier (Table 2) (49). We considered significant changes in the apparent *K*_D_ of more than twofold from unmodified IgG to be potentially meaningful changes and within the scope of the SPR method, using a simplified 1:1 Langmuir model that does not fully represent the actual interaction which is more complicated (39). No significant effects of glycan changes above twofold were seen on the binding to FcγRIa, FcγRIIa (neither the H131- or the R131-allotype), or FcγRIIb/c (Figures 2A–D). However, marked changes were seen for all FcγRIII-isoforms. Reduction of fucose resulted in enhanced binding to all FcγRIII species by approximately 10- to 20-fold depending on the type- and allotype (Figures 2E–H), as reported (4, 5). Importantly, addition of galactose consistently enhanced binding of hypo-fucosylated IgG1 for all FcγRIIIa allotypes, doubling the effect of hypo-fucosylation alone (Figures 2E,F). This effect was also seen for allotypes of FcγRIIIb, but less strong and only for IgG1 that was bisected in addition to hypo-fucosylated (Figures 2G,H). Further sialylation of low-fucosylated galactosylated IgG1 had little additional effect on the binding to FcγRIII, except for hypo-fucosylated and bisected IgG1 for both allotypes of FcγRIIIa and FcγRIIIb NA2, where sialylation cause a significant decrease in binding. Taken together, glycan changes in the IgG-Fc only affect binding to FcγRIIIa and FcγRIIIb, with a major effect of hypo-fucosylation increasing binding to FcγRIIIa/b that was boosted by galactosylation. Bisection only appeared to indirectly affect binding when occurring in conjunction with sialylation, causing a slight decreased binding to FcγRIIIa/b to otherwise hypo-fucosylated and galactosylated IgG.

**Table 2:** Affinity of unmodified IgG1 (U) to the different FcγRs.

	Mean affinity	SEM
FcγRI	3.0 × 10^−9^	±8.7 × 10^−10^
FcγRIIa^131H^	3.8 × 10^−7^	±1.3 × 10^−8^
FcγRIIa^131R^	4.8 × 10^−7^	±2.8 × 10^−8^
FcγRIIb	2.7 × 10^−6^	±1.1 × 10^−7^
FcγRIIIa^158F^	1.3 × 10^−6^	±9.5 × 10^−8^
FcγRIIIa^158V^	2.4 × 10^−7^	±1.0 × 10^−8^
FcγRIIIb NA1	3.2 × 10^−6^	±4.7 × 10^−7^
FcγRIIIb NA2	2.8 × 10^−6^	±1.0 × 10^−7^

*Affinity in K_D_, as measured by SPR*.

### FcγRIIIa-Mediated ADCC Is Steered by Fucosylation and Galactosylation

**Figure 3:**
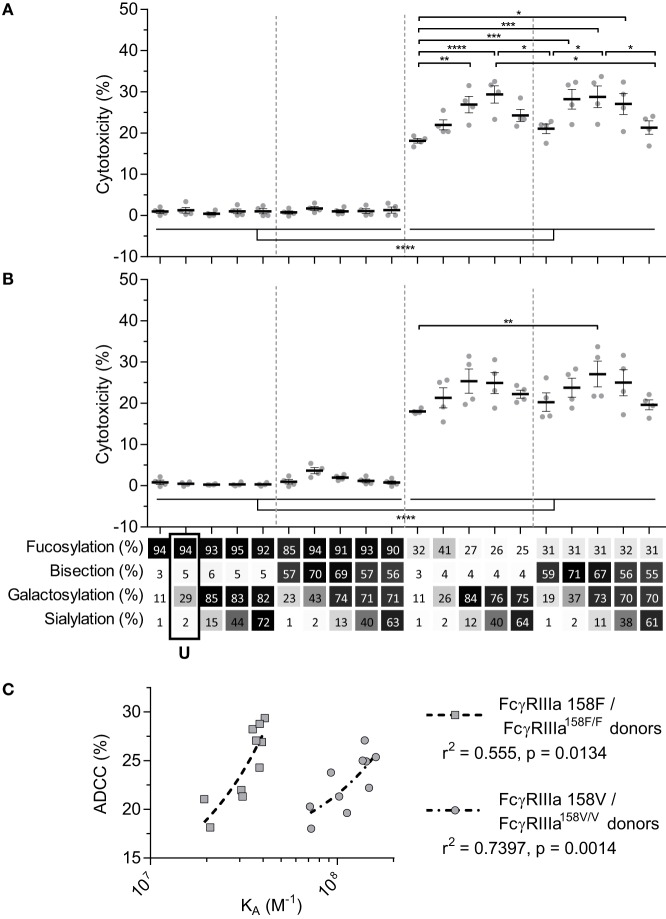
NK cell-mediated antibody-dependent cellular cytotoxicity (ADCC) of anti-D glycoform opsonized red blood cell. ADCC mediated by NK cells from monozygotic FcγRIIIA^158F/F^ donors **(A)**, or monozygotic FcγRIIIA^158V/V^ donors **(B)**, data represent the mean and SEM of four combined independent experiments; *, **, ***, and **** denote a statistical significance of *p* ≤ 0.05, *p* ≤ 0.01, *p* ≤ 0.001, and *p* ≤ 0.0001, respectively, as tested by one-way ANOVA using Tukey’s multiple comparisons test. *x*-Axis legend describes the percentage of each derived glycan trait indicated and by grayscale, from light to dark. **(C)** Correlation between *K*_A_ of FcγRIIIa F158 or FcγRIIIa V158 binding of hypo-fucosylated glycoforms and ADCC activity of FcγRIIIA^158F/F^ or FcγRIIIA^158V/V^ donors, respectively. *r*^2^ and *p* value shown where obtained using a two-tailed Pearson’s correlation. U: unmodified glycoform.

We next tested the efficacy of these anti-D IgG1 antibodies to mediate ADCC against RBC. Curiously, no NK cell-mediated induction of ADCC was seen with any fucosylated IgG1 at any concentration tested (Figures 3A,B; Figure S3 in Supplementary Material). Only hypo-fucosylated IgG1 induced ADCC in variable degrees depending on the glycosylation (Figures 3A,B). The observed level of ADCC were in line with the binding results obtained by SPR for each of the FcγRIIIa allotypes (Figure 3C), confirming the essential role of both hypo-fucosylation and elevated galactosylation for increased FcγRIIIa-binding and effector functions. Again, sialic acid had a minor but significant negative effect, especially for the bisected, hypo-fucosylated, and galactosylated IgG1 (Figures 3A,B). Remarkably, the well-known allotypic differences in affinity were confirmed by our SPR experiments, but not by the functional NK cell-mediated ADCC.

### Galactosylation and Sialylation Direct Complement Binding and Activation

**Figure 4:**
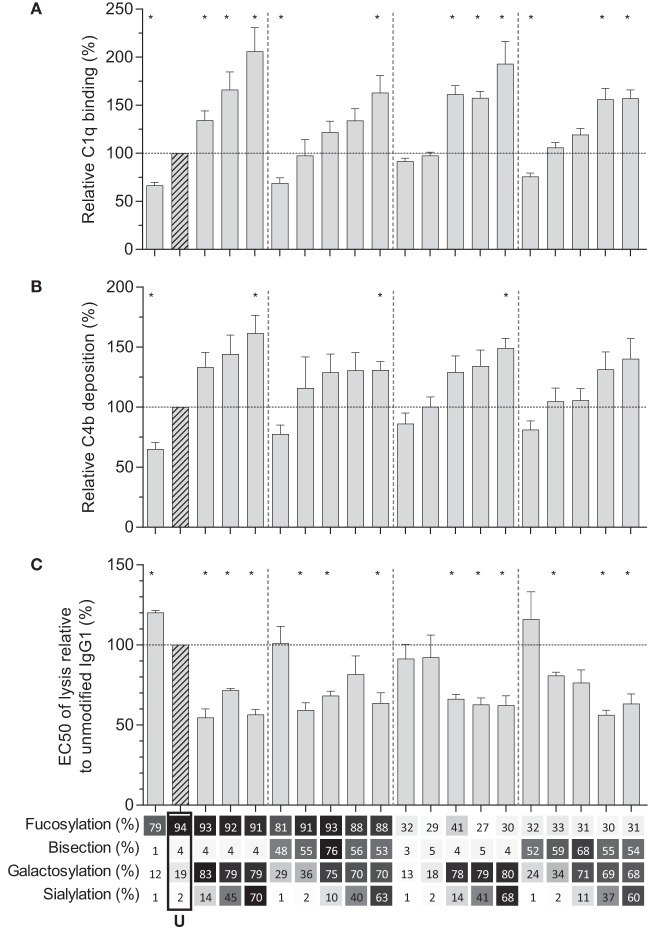
Complement activation by glyco-engineered anti-TNP IgG1. Relative **(A)** binding of C1q (*n* = 4) and **(B)** C4 deposition as determined by ELISA (*n* = 4), **(C)** complement-mediated lysis of aTNP opsonized red blood cells (*n* = 3). Data represent the mean and SEM of combined independent experiments; *denotes a statistical significance of *p* ≤ 0.05, as tested by a one-sample *t*-test against a theoretical mean of 100 (%). *x*-Axis legend describes the percentage of each derived glycan trait indicated and by grayscale, from light to dark. U: unmodified glycoform.

**Figure 5:**
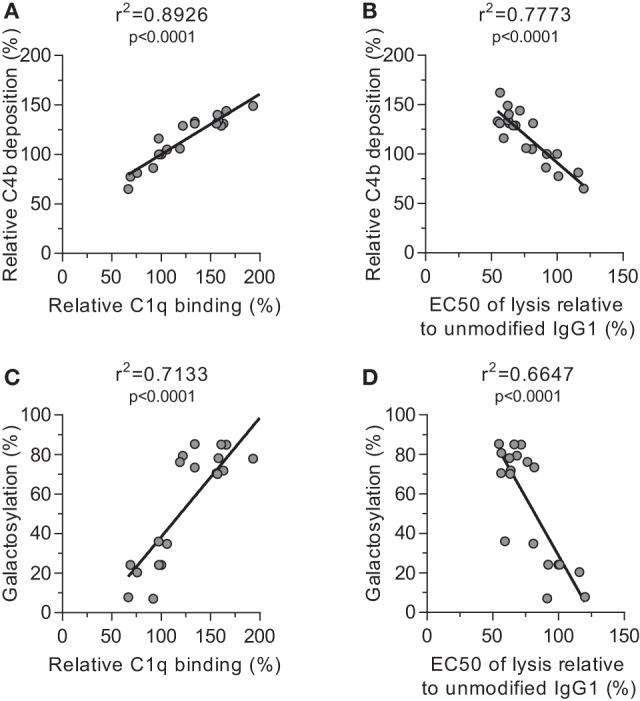
Correlations between complement activation and galactosylation. Correlation between **(A)** galactosylation and C1q binding, **(B)** C1q binding and C4 deposition, **(C)** C4 deposition and complement-mediated red blood cell lysis, and **(D)** galactosylation and lysis, statistically tested using a two-tailed Pearson’s correlation.

We then tested the effect of IgG-Fc glycosylation on C1q binding and subsequent complement activation, using the anti-TNP panel of IgG1 antibodies as anti-D does not fix complement. The efficiency of C1q binding to TNP-lated human serum albumin (TNP-HSA) and subsequent C4b deposition was titrated by serial dilution (Figure S4 in Supplementary Material). All glycovariants of anti-TNP bound TNP-HSA equally well (Figure S4A in Supplementary Material), but C1q binding and C4b deposition differed profoundly for the different glycoforms (Figure S4B in Supplementary Material). The relative C1q binding and C4b deposition were then calculated (Figures 4A,B, respectively). Both data sets suggested that elevated galactosylation and sialylation positively influenced complement activity. This activity was fully depended on the classical pathway with no influence of the mannan-binding lectin- or the alternative pathway, as C4b and C3b deposition, were completely blocked by an anti-C1q blocking antibody (Figure S5 in Supplementary Material). We then determined if this also translates into more efficient complement-dependent cytotoxicity (CDC) by analyzing complement-dependent lysis of TNP-labeled RBC (Figure 4C; Figure S6 in Supplementary Material). The level of C1q binding of each glycoform correlated well with the C4b deposition (Figure 5A) and with the obtained EC_50_ of CDC (Figure 5B). The level of galactosylation of each glycoform also showed a direct relationship with the efficacy of C1q binding, and EC_50_ (Figures 5C,D). In conclusion, the degree of galactosylation, but also sialylation of the IgG1-Fc N-glycan directly steers the antibody’s efficacy to stimulate complement deposition and CDC.

## Discussion

We have previously created an orthogonal set of glyco-engineering tools (31) which we now combined to create 20 glycovariants of human IgG1, representing natural variants found in human plasma IgG, including extreme glycoforms found for examples in patients with FNAIT and HDFN (17, 18, 21, 22). These variants were investigated for their functional capacity to engage and activate FcγR and complement.

Of the FcγRs, we only observed an effect of glycosylation on binding to the FcγRIII-family of receptors, both FcγRIIIa and FcγRIIIb and their allotypes, which confirms and expands recent studies using a limited set of glycovariants presented here (24, 30). Increased FcγRIII binding seems to be a general phenomenon for all IgG subclasses upon afucosylation (50, 51). The positive binding effects were primarily caused by the lack of fucose, which was further strengthened by additional galactose. A similar effect has been observed for neutralizing anti-HIV antibody 2G12 produced in modified plant cells which showed better FcγRIIIa binding and antibody-dependent (NK) cell-mediated viral inhibition (52).

The enhanced binding of galactosylated and afucosylated IgG was slightly weakened by addition of sialic acid, but only if a bisecting GlcNAc was present. A similar negative effect of sialylation has previously been observed for mouse FcγR by Ravetch and colleagues (53). Importantly, we showed that the enhanced FcγRIII-binding effects are directly translated into increased FcγR-mediated cellular functions. We tested this using NK cell-mediated ADCC, as NK cells are the only cell type that only express FcγRIIIa. Curiously, we observed no ADCC at all for fucosylated IgG, even at high concentrations of IgG1. Thus, ADCC activity was only observed with afucosylated IgG1. Although somewhat surprising, this phenomenon has been observed previously for anti-Rhesus-mediated ADCC (54), but also for Rituximab-mediated B cell killing (27). This suggests that the enhanced affinity afucosylation of IgG has on FcγRIIIa binding is required to cross a signaling threshold of FcγRIIIa on NK cells required for killing.

The second surprise was that no significant difference was observed between ADCC-capacity of NK cells from donors homozygous for one of the two FcγRIIIa-V/F158 allotypes, of which the V158 allele is known to have higher affinity for IgG (also confirmed here to be ~2–5×) (49). *In vitro*, this has been found result in stronger functional efficacy for the V158-variant (55–57). *In vivo*, conflicting reports have showed that individuals homozygous either the V158 or the F158 allotype show stronger cellular clearance (58–61). It should be noted that most of these studies were performed before the knowledge of FcγRIII gene being influenced by copy number variation (61). We also now know that NK cells can also express FcγRIIc or FcγRIIb in some individuals. Both these variations affect the functionality of this receptor (32, 62, 63). In this study, we eliminated both these variables by selecting donors with two copies of FcγRIIIa and without FcγRIIc-ORF, possibly explaining these discrepancies, and perhaps suggesting that the twofold to fivefold difference in affinity of IgG1 allotype is not enough to cause functional differences.

Importantly, the observed changes in FcγRIIIa-binding due to glycosylation reliably translated into functional NK cell-mediated ADCC lysis of RBC. For FcγRIIIa and FcγRIIIb it was known that absence of IgG-Fc core-fucosylation increases the affinity of interaction due to a glycan–glycan interaction between the Fc glycan and the N162-glycan uniquely found in the FcγRIII family (11). Our approach to combine this with multiple end glycan editing shows an additional layer of complexity exerted by the galactose and sialic acid. The reasons for this added effect of galactose is unknown but may very well be related to the subtle effects on quaternary structure of the Fc-domain (64, 65) but may also be related to differential interaction of the Fc-glycan with the N162-glycan found in FcγRIII (11).

The possible effect of the Fc-glycans on complement activity, has until now remained enigmatic. It has been proposed for a long time that agalactosylated IgG activates complement more efficiently through the lectin pathway (MBL) (25). To our knowledge these results have never been confirmed. On the contrary, we saw enhanced complement activity of all glycovariants with elevated galactose, and no evidence of MBL being activated by any of our glycoforms. These results confirm recent work also suggesting galactosylation of IgG1 to positively influence C1q binding and CDC (26, 66). In addition, our results clearly rule out fucosylation or bisection having an effect on complement activation, and we now show that sialylation increases the C1q-binding of galactosylated IgG. This effect of sialylation was observed on all different glycan backbones (e.g., with or without fucose, with or without bisection) which is highly suggestive that this is no artifactual finding. This is in contrast with the previously mentioned study showing that additional sialylation decreases C1q binding (26). Activation of complement is dependent on spatial arrangement of the IgG on the cell surface (67) which is likely to differ considerably between each monoclonal antibody and target, and may possibly explain the discrepancies found between our two studies. This view is supported by our observations that sialylation had limited if any effect on IgG-mediated CDC using RBC as targets, while binding to C1q of anti-TNP antibodies was enhanced by sialylated IgG on solid surfaces.

Low galactosylation level in total IgG generally correlates with disease severity of several autoimmune diseases, such as rheumatoid arthritis and multiple sclerosis (13, 14). While this may seem at odds with our observations at first glance, with high galactosylated IgG having elevated complement and FcγR activities, both notions are in agreement if the balance between total- and antigen-specific glycosylation is taken into account. In this way, low potential for FcγR- and C1q binding for total IgG (e.g., low galactosylation), creates a pro-inflammatory environment in which clinical manifestations can take hold as this lowers the threshold for pathogenic antibodies. Antigen-specific IgG can also potentially have different glycosylation features than total IgG as we have shown before (17, 18, 21, 22), and if these are more pro-inflammatory than that of total IgG, this can theoretically lead to enhanced immune activation and clinical symptoms. The knowledge obtained in the current research provides a roadmap to decipher the meaning of glycan profiles in these diseases settings.

**Figure 6:**
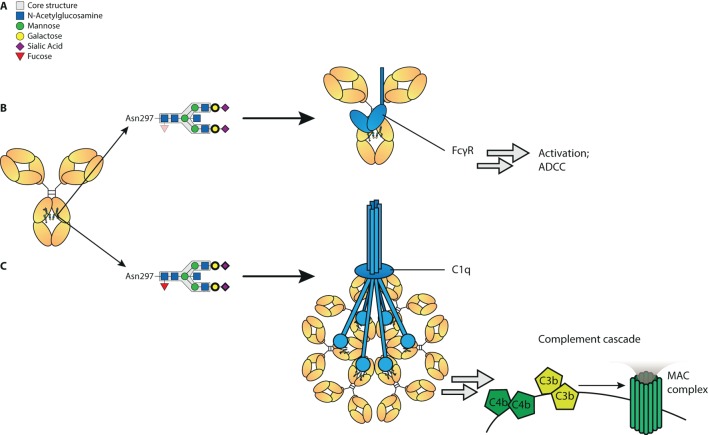
Proposed model of influence of immunoglobulin G (IgG)-Fc glycan composition on effector functions. **(A)** Standard composition of bi-antennary Fc-glycan, **(B)** Afucosylation of IgG-Fc glycan increases binding affinity to FcγRIII and subsequent antibody-mediated functions, such as antibody-dependent cellular cytotoxicity (ADCC). In addition galactosylation further increases affinity to FcγRIII and function of afucosylated IgG. **(C)** Galactosylation enhances binding of IgG to complement component C1q and activation of the classical complement pathway, which results in cleavage of complements C4, C3, and further initiation of the membrane attack complex (MAC). Sialylation may further increase C1q binding and complement activation. Glycan residues that need to be present to enhance indicated effector function (ADCC/complement-dependent cytotoxicity) are displayed with bolder lines, and for those that need to be absent to enhance indicated effector functions are displayed with faded colors.

In summary, we show here that a set of glyco-engineering techniques we recently developed (31) can be combined to quickly generate any desired IgG glycoforms to test the effect on functional capacity. Using two sets of monoclonal antibodies we generated the most extreme 20 different glycoforms possible, and examined their effect on binding to FcγR and complement, as well as their functional capacity to trigger cytotoxicity. These revealed first that the normal glycosylation changes seen in human IgG1 do not affect any other FcγR than FcγRIIIa and FcγRIIIb. Second, hypo-fucosylation and galactosylation increase binding to both human FcγRIII-variants, with a minor negative effect of sialic acid and bisecting GlcNAc. In addition, galactosylation is the primary glycan adduct that enhances C1q-binding and all downstream complement activities, including CDC. This is summarized in Figure 6. Collectively, this indicates that afucosylated and hyper-galactosylated IgG1 antibodies have both improved ADCC and complement-mediated activities, including complement opsonization and CDC. These properties can now be systematically implemented in new therapeutic antibodies for enhanced effector functions. Even as important, this also allows us to decipher the clinical potency of antibodies in immune responses that have tendency to have altered fucosylation and/or galactosylation (17, 18, 21, 22).

## Ethics Statement

Peripheral blood from anonymous, healthy volunteers was obtained with informed, written consent of all subjects, in accordance with Dutch regulations. This study was approved by the Sanquin Ethical Advisory Board in accordance with the Declaration of Helsinki.

## Author Contributions

GD, TK, DW, TR, MW, and GV designed the research. GD, AB, DW, TR, MW, and GV designed the experiments. GD, LT, RP, AB, MdB, CK, SL-T, RV, and YM performed the experiments. GD, RP, AB, MdB, CK, TR, TK, MW, and GV analyzed data, GD and GV wrote the manuscript. All authors contributed to and approved the final manuscript.

## Conflict of Interest Statement

The authors declare that this study received funding from Sanquin Bloedvoorziening, a not-for-profit organization. The funder was not involved in the study design or collection, analysis, or interpretation of the data.

## References

[1] Bruhns P Jönsson F. Mouse and human FcR effector functions. Immunol Rev (2015) 268:25–51. 10.1111/imr.12350 26497511

[2] Weiner LM Surana R Wang S. Monoclonal antibodies: versatile platforms for cancer immunotherapy. Nat Rev Immunol (2010) 10:317–27. 10.1038/nri2744 20414205 PMC3508064

[3] Weiner GJ. Building better monoclonal antibody-based therapeutics. Nat Rev Cancer (2015) 15:361–70. 10.1038/nrc3930 25998715 PMC4491443

[4] Shields RL Lai J Keck R O’Connell LY Hong K Meng YG Lack of fucose on human IgG1 N-linked oligosaccharide improves binding to human Fcgamma RIII and antibody-dependent cellular toxicity. J Biol Chem (2002) 277:26733–40. 10.1074/jbc.M202069200 11986321

[5] Shinkawa T Nakamura K Yamane N Shoji-Hosaka E Kanda Y Sakurada M The absence of fucose but not the presence of galactose or bisecting N-acetylglucosamine of human IgG1 complex-type oligosaccharides shows the critical role of enhancing antibody-dependent cellular cytotoxicity. J Biol Chem (2003) 278:3466–73. 10.1074/jbc.M210665200 12427744

[6] Beck A Reichert JM. Marketing approval of mogamulizumab: a triumph for glyco-engineering. MAbs (2012) 4:419–25. 10.4161/mabs.20996 22699226 PMC3499336

[7] Subedi GP Hanson QM Barb AW. Restricted motion of the conserved immunoglobulin G1 N-glycan is essential for efficient FcγRIIIa binding. Structure (2014) 22:1478–88. 10.1016/j.str.2014.08.002 25199692 PMC4192013

[8] Jefferis R. Recombinant antibody therapeutics: the impact of glycosylation on mechanisms of action. Trends Pharmacol Sci (2009) 30:356–62. 10.1016/j.tips.2009.04.007 19552968

[9] Caaveiro JMM Kiyoshi M Tsumoto K. Structural analysis of Fc/FcγR complexes: a blueprint for antibody design. Immunol Rev (2015) 268:201–21. 10.1111/imr.12365 26497522

[10] Sondermann P Huber R Oosthuizen V Jacob U. The 3.2-A crystal structure of the human IgG1 Fc fragment-Fc gammaRIII complex. Nature (2000) 406:267–73. 10.1038/35018508 10917521

[11] Ferrara C Grau S Jäger C Sondermann P Brünker P Waldhauer I Unique carbohydrate-carbohydrate interactions are required for high affinity binding between FcgammaRIII and antibodies lacking core fucose. Proc Natl Acad Sci U S A (2011) 108:12669–74. 10.1073/pnas.1108455108 21768335 PMC3150898

[12] Baković MP Selman MHJ Hoffmann M Rudan I Campbell H Deelder AM High-throughput IgG Fc N-glycosylation profiling by mass spectrometry of glycopeptides. J Proteome Res (2013) 12:821–31. 10.1021/pr300887z 23298168

[13] Bondt A Selman MHJ Deelder AM Hazes JMW Willemsen SP Wuhrer M Association between galactosylation of immunoglobulin G and improvement of rheumatoid arthritis during pregnancy is independent of sialylation. J Proteome Res (2013) 12:4522–31. 10.1021/pr400589m 24016253

[14] Wuhrer M Selman MHJ McDonnell LA Kümpfel T Derfuss T Khademi M Pro-inflammatory pattern of IgG1 Fc glycosylation in multiple sclerosis cerebrospinal fluid. J Neuroinflammation (2015) 12:235. 10.1186/s12974-015-0450-1 26683050 PMC4683913

[15] Chen G Wang Y Qiu L Qin X Liu H Wang X Human IgG Fc-glycosylation profiling reveals associations with age, sex, female sex hormones and thyroid cancer. J Proteomics (2012) 75:2824–34. 10.1016/j.jprot.2012.02.001 22365975

[16] Wuhrer M Porcelijn L Kapur R Koeleman CAM Deelder A de Haas M Regulated glycosylation patterns of IgG during alloimmune responses against human platelet antigens. J Proteome Res (2009) 8:450–6. 10.1021/pr800651j 18942870

[17] Kapur R Della Valle L Sonneveld M Hipgrave Ederveen A Visser R Ligthart P Low anti-RhD IgG-Fc-fucosylation in pregnancy: a new variable predicting severity in haemolytic disease of the fetus and newborn. Br J Haematol (2014) 166:936–45. 10.1111/bjh.12965 24909983 PMC4282073

[18] Sonneveld ME Natunen S Sainio S Koeleman CAM Holst S Dekkers G Glycosylation pattern of anti-platelet IgG is stable during pregnancy and predicts clinical outcome in alloimmune thrombocytopenia. Br J Haematol (2016) 174:310–20. 10.1111/bjh.14053 27017954

[19] Ackerman ME Crispin M Yu X Baruah K Boesch AW Harvey DJ Natural variation in Fc glycosylation of HIV-specific antibodies impacts antiviral activity. J Clin Invest (2013) 123:2183–92. 10.1172/JCI65708 23563315 PMC3637034

[20] Wang TT Sewatanon J Memoli MJ Wrammert J Bournazos S Bhaumik SK IgG antibodies to dengue enhanced for FcγRIIIA binding determine disease severity. Science (2017) 355:395–8. 10.1126/science.aai8128 28126818 PMC5557095

[21] Kapur R Kustiawan I Vestrheim A Koeleman CA Visser R Einarsdottir HK A prominent lack of IgG1-Fc fucosylation of platelet alloantibodies in pregnancy. Blood (2014) 123:471–80. 10.1182/blood-2013-09-527978 24243971 PMC3901064

[22] Sonneveld ME Koelewijn J de Haas M Admiraal J Plomp R Koeleman CAM Antigen specificity determines anti-red blood cell IgG-Fc alloantibody glycosylation and thereby severity of haemolytic disease of the fetus and newborn. Br J Haematol (2017) 176:651–60. 10.1111/bjh.14438 27891581

[23] Kapur R Heitink-poll KMJ Porcelijn L Bentlage AEH Bruin MC Visser R C-reactive protein enhances IgG-mediated phagocyte responses and thrombocytopenia. Blood (2015) 125:1793–803. 10.1182/blood-2014-05-579110 25548320

[24] Thomann M Schlothauer T Dashivets T Malik S Avenal C Bulau P In vitro glycoengineering of IgG1 and its effect on Fc receptor binding and ADCC activity. PLoS One (2015) 10:e0134949. 10.1371/journal.pone.0134949 26266936 PMC4534130

[25] Malhotra R Wormald MR Rudd PM Fischer PB Dwek RA Sim RB. Glycosylation changes of IgG associated with rheumatoid arthritis can activate complement via the mannose-binding protein. Nat Med (1995) 1:237–43. 10.1038/nm0395-237 7585040

[26] Quast I Keller CW Maurer MA Giddens JP Tackenberg B Wang LX Sialylation of IgG Fc domain impairs complement-dependent cytotoxicity. J Clin Invest (2015) 125:4160–70. 10.1172/JCI82695 26436649 PMC4639970

[27] Li H Sethuraman N Stadheim TA Zha D Prinz B Ballew N Optimization of humanized IgGs in glycoengineered *Pichia pastoris*. Nat Biotechnol (2006) 24:210–5. 10.1038/nbt1178 16429149

[28] Yang Z Wang S Halim A Schulz MA Frodin M Rahman SH Engineered CHO cells for production of diverse, homogeneous glycoproteins. Nat Biotechnol (2015) 33:2014–7. 10.1038/nbt.3280 26192319

[29] Meuris L Santens F Elson G Festjens N Boone M Dos Santos A GlycoDelete engineering of mammalian cells simplifies N-glycosylation of recombinant proteins. Nat Biotechnol (2014) 32:485–9. 10.1038/nbt.2885 24752077 PMC7039703

[30] Subedi GP Barb AW The immunoglobulin G1 N-glycan composition affects binding to each low affinity Fc γ receptor. MAbs (2016) 8:1512–24. 10.1080/19420862.2016.1218586 PMC5098437 27492264

[31] Dekkers G Plomp R Koeleman CAM Visser R von Horsten HH Sandig V Multi-level glyco-engineering techniques to generate IgG with defined Fc-glycans. Sci Rep (2016) 6:36964. 10.1038/srep36964 27872474 PMC5131652

[32] van der Heijden J Breunis WB Geissler J de Boer M van den Berg TK Kuijpers TW. Phenotypic variation in IgG receptors by nonclassical FCGR2C alleles. J Immunol (2012) 188:1318–24. 10.4049/jimmunol.1003945 22198951

[33] Della Valle L Dohmen SE Verhagen OJHM Berkowska MA Vidarsson G Ellen van der Schoot C. The majority of human memory B cells recognizing RhD and tetanus resides in IgM+ B cells. J Immunol (2014) 193:1071–9. 10.4049/jimmunol.1400706 24965774 PMC4105240

[34] Kruijsen D Einarsdottir HK Schijf MA Coenjaerts FE van der Schoot EC Vidarsson G Intranasal administration of antibody-bound respiratory syncytial virus particles efficiently primes virus-specific immune responses in mice. J Virol (2013) 87:7550–7. 10.1128/JVI.00493-13 23637394 PMC3700286

[35] Chambers MC Maclean B Burke R Amodei D Ruderman DL Neumann S A cross-platform toolkit for mass spectrometry and proteomics. Nat Biotechnol (2012) 30:918–20. 10.1038/nbt.2377 23051804 PMC3471674

[36] Plomp R Dekkers G Rombouts Y Visser R Koeleman CAM Kammeijer GSM Hinge-region O-glycosylation of human immunoglobulin G3 (IgG3). Mol Cell Proteomics (2015) 14:1373–84. 10.1074/mcp.M114.047381 25759508 PMC4424406

[37] Ory PA Clark MR Kwoh EE Clarkson SB Goldstein IM. Sequences of complementary DNAs that encode the NA1 and NA2 forms of Fc receptor III on human neutrophils. J Clin Invest (1989) 84:1688–91. 10.1172/JCI114350 2478590 PMC304039

[38] Rodenko B Toebes M Hadrup SR van Esch WJE Molenaar AM Schumacher TNM Generation of peptide-MHC class I complexes through UV-mediated ligand exchange. Nat Protoc (2006) 1:1120–32. 10.1038/nprot.2006.121 17406393

[39] Dekkers G Bentlage AEH Stegmann TC Howie HL Lissenberg-Thunnissen S Zimring J Affinity of human IgG subclasses to mouse Fc gamma receptors. MAbs (2017) 1–7. 10.1080/19420862.2017.1323159 PMC5524164 28463043

[40] de Lau W Barker N Low TY Koo B-K Li VSW Teunissen H Lgr5 homologues associate with Wnt receptors and mediate R-spondin signalling. Nature (2011) 476:293–7. 10.1038/nature10337 21727895

[41] Schasfoort RBM Andree KC van der Velde N van der Kooi A Stojanović I Terstappen LWMM. Interpolation method for accurate affinity ranking of arrayed ligand-analyte interactions. Anal Biochem (2016) 500:21–3. 10.1016/j.ab.2016.01.023 26878776

[42] McGrath FDG Brouwer MC Arlaud GJ Daha MR Hack CE Roos A. Evidence that complement protein C1q interacts with C-reactive protein through its globular head region. J Immunol (2006) 176:2950–7. 10.4049/jimmunol.176.5.2950 16493053

[43] Leito JTD Ligtenberg AJM van Houdt M van den Berg TK Wouters D. The bacteria binding glycoprotein salivary agglutinin (SAG/gp340) activates complement via the lectin pathway. Mol Immunol (2011) 49:185–90. 10.1016/j.molimm.2011.08.010 21920605

[44] Hack CE Paardekooper J Smeenk RJ Abbink J Eerenberg AJ Nuijens JH. Disruption of the internal thioester bond in the third component of complement (C3) results in the exposure of neodeterminants also present on activation products of C3. An analysis with monoclonal antibodies. J Immunol (1988) 141:1602–9. 2457622

[45] Armitage P Colton T In: Armitage P Colton Chichester T, editors. Encyclopedia of Biostatistics. UK: John Wiley & Sons, Ltd (2005).

[46] Fokkink WJR Falck D Santbergen TCM Huizinga R Wuhrer M Jacobs BC. Comparison of Fc N-glycosylation of pharmaceutical products of intravenous immunoglobulin G. PLoS One (2015) 10:e0139828. 10.1371/journal.pone.0139828 26457892 PMC4601728

[47] Barb AW Brady EK Prestegard JH. Branch-specific sialylation of IgG-Fc glycans by ST6Gal-I. Biochemistry (2009) 48:9705–7. 10.1021/bi901430h 19772356 PMC2761508

[48] Washburn N Schwab I Ortiz D Bhatnagar N Lansing JC Medeiros A Controlled tetra-Fc sialylation of IVIg results in a drug candidate with consistent enhanced anti-inflammatory activity. Proc Natl Acad Sci U S A (2015) 112:201422481 10.1073/pnas.1422481112 PMC4371931 25733881

[49] Bruhns P Iannascoli B England P Mancardi DA Fernandez N Jorieux S Specificity and affinity of human Fcgamma receptors and their polymorphic variants for human IgG subclasses. Blood (2009) 113:3716–25. 10.1182/blood-2008-09-179754 19018092

[50] Niwa R Natsume A Uehara A Wakitani M Iida S Uchida K IgG subclass-independent improvement of antibody-dependent cellular cytotoxicity by fucose removal from Asn297-linked oligosaccharides. J Immunol Methods (2005) 306:151–60. 10.1016/j.jim.2005.08.009 16219319

[51] Bruggeman CW Dekkers G Bentlage AEH Treffers LW Nagelkerke SQ Lissenberg-Thunnissen S Enhanced effector functions due to antibody defucosylation depend on the effector cell Fcγ receptor profile. J Immunol (2017) 199(1):204–11. 10.4049/jimmunol.1700116 28566370

[52] Forthal DN Gach JS Landucci G Jez J Strasser R Kunert R Fc-glycosylation influences Fcγ receptor binding and cell-mediated anti-HIV activity of monoclonal antibody 2G12. J Immunol (2010) 185:6876–82. 10.4049/jimmunol.1002600 21041724

[53] Kaneko Y Nimmerjahn F Ravetch JV. Anti-inflammatory activity of immunoglobulin G resulting from Fc sialylation. Science (2006) 313:670–3. 10.1126/science.1129594 16888140

[54] Sibéril S de Romeuf C Bihoreau N Fernandez N Meterreau J-L Regenman A Selection of a human anti-RhD monoclonal antibody for therapeutic use: impact of IgG glycosylation on activating and inhibitory Fc gamma R functions. Clin Immunol (2006) 118:170–9. 10.1016/j.clim.2005.10.008 16332457

[55] López-Albaitero A Lee SC Morgan S Grandis JR Gooding WE Ferrone S Role of polymorphic Fc gamma receptor IIIa and EGFR expression level in cetuximab mediated, NK cell dependent in vitro cytotoxicity of head and neck squamous cell carcinoma cells. Cancer Immunol Immunother (2009) 58:1853–64. 10.1007/s00262-009-0697-4 19319529 PMC3426289

[56] Hatjiharissi E Xu L Santos DD Hunter ZR Ciccarelli BT Verselis S Increased natural killer cell expression of CD16, augmented binding and ADCC activity to rituximab among individuals expressing the Fc{gamma}RIIIa-158 V/V and V/F polymorphism. Blood (2007) 110:2561–4. 10.1182/blood-2007-01-070656 17475906 PMC1988936

[57] Oboshi W Watanabe T Matsuyama Y Kobara A Yukimasa N Ueno I The influence of NK cell-mediated ADCC: structure and expression of the CD16 molecule differ among FcγRIIIa-V158F genotypes in healthy Japanese subjects. Hum Immunol (2016) 77:165–71. 10.1016/j.humimm.2015.11.001 26582002

[58] Stegmann TC Veldhuisen B Nagelkerke SQ Winkelhorst D Schonewille H Verduin EP RhIg-prophylaxis is not influenced by FCGR2/3 polymorphisms involved in red blood cell clearance. Blood (2017) 129:1045–8. 10.1182/blood-2016-05-716365 28082442

[59] Cartron G Dacheux L Salles G Solal-Celigny P Bardos P Colombat P Therapeutic activity of humanized anti-CD20 monoclonal antibody and polymorphism in IgG Fc receptor FcgammaRIIIa gene. Blood (2002) 99:754–8. 10.1182/blood.V99.3.754 11806974

[60] Burkhardt B Yavuz D Zimmermann M Schieferstein J Kabickova E Attarbaschi A Impact of Fc gamma-receptor polymorphisms on the response to rituximab treatment in children and adolescents with mature B cell lymphoma/leukemia. Ann Hematol (2016) 95:1503–12. 10.1007/s00277-016-2731-x 27376362

[61] Kumpel BM De Haas M Koene HR Van De Winkel JGJ Goodrick MJ. Clearance of red cells by monoclonal IgG3 anti-D in vivo is affected by the VF polymorphism of Fcgamma RIIIa (CD16). Clin Exp Immunol (2003) 132:81–6. 10.1046/j.1365-2249.2003.02119.x 12653840 PMC1808672

[62] Aitman TJ Dong R Vyse TJ Norsworthy PJ Johnson MD Smith J Copy number polymorphism in Fcgr3 predisposes to glomerulonephritis in rats and humans. Nature (2006) 439:851–5. 10.1038/nature04489 16482158

[63] Thabet MM Huizinga TWJ Marques RB Stoeken-Rijsbergen G Bakker AM Kurreeman FA Contribution of Fcgamma receptor IIIA gene 158V/F polymorphism and copy number variation to the risk of ACPA-positive rheumatoid arthritis. Ann Rheum Dis (2009) 68:1775–80. 10.1136/ard.2008.099309 19019892

[64] Ahmed AA Giddens J Pincetic A Lomino JV Ravetch JV Wang L-X Structural characterization of anti-inflammatory immunoglobulin G Fc proteins. J Mol Biol (2014) 426:3166–79. 10.1016/j.jmb.2014.07.006 25036289 PMC4159253

[65] Le NPL Bowden TA Struwe WB Crispin M. Immune recruitment or suppression by glycan engineering of endogenous and therapeutic antibodies. Biochim Biophys Acta (2016) 1860:1655–68. 10.1016/j.bbagen.2016.04.016 27105835 PMC4922387

[66] Pace D Lewis N Wu T Gillespie R Leiske D Velayudhan J Characterizing the effect of multiple Fc glycan attributes on the effector functions and FcγRIIIa receptor binding activity of an IgG1 antibody. Biotechnol Prog (2016) 32(5):1181–92. 10.1002/btpr.2300 27160519

[67] Diebolder CA Beurskens FJ de Jong RN Koning RI Strumane K Lindorfer MA Complement is activated by IgG hexamers assembled at the cell surface. Science (2014) 343:1260–3. 10.1126/science.1248943 24626930 PMC4250092

