# Supplementary material for: Decoding the Human Immunoglobulin G-Glycan Repertoire Reveals a Spectrum of Fc-Receptor- and Complement-Mediated-Effector Activities
Source: Front Immunol. 2017 Aug 2;8:877. doi: 10.3389/fimmu.2017.00877 (PMC5539844; doi:10.3389/fimmu.2017.00877)
Supplement: Supplementary file 1 [file Data_Sheet_1.PDF]

Supplementary Material

Decoding the human IgG-glycan repertoire reveals a spectrum of Fc-receptor- and complement-mediated- effector activities

Gillian Dekkers, Louise Treffers, Rosina Plomp, Arthur E.H. Bentlage, Marcella de Boer, Carolien A.M. Koeleman, Suzanne Lissenberg-Thunnissen, Remco Visser, Mieke Brouwer, Juk Yee Mok, Hanke Matlung, Timo van den Berg, Wim J.E. van Esch, Taco Kuijpers, Diana Wouters, Theo Rispens, Manfred Wuhrer and Gestur Vidarsson*

*** Correspondence:** Gestur Vidarsson: G.Vidarsson@sanquin.nl

1 Supplementary Figures and Tables

1.1 Supplementary Table 1

Comprehensive list of glycopeptide degrees of non-complex glycans found in the glyco-engineered IgG1 batches of anti-D and anti-TNP specificity.

1.1 Supplementary table 1

Comprehensive list of glycopeptide degrees of non-complex glycans found in the glyco-engineered IgG1 batches of anti-D and anti-TNP specificity.

name	glyco-engineering						relative abundance glycopeptides*																										
**anti-D**	2FF (-F)	2FG (-G)	GNT3 (+B)	B4GALT1/D-galactose (+G)	ST6GALT (+S)	in vitro sialylation (+ivs)	H5N3F1	H6N3F1	H6N4F1	H6N5F1	H5N3F1S1	H6N3F1S1	H6N4F1S1	H6N5F1S1	H5N3	H6N3	H6N4	H6N5	H5N3S1	H6N3S1	H6N4S1	H6N5S1	H3N3F1	H4N3F1	H4N3F1S1	H3N3	H4N3	H4N3S1	H5N2	H6N2	H7N2	H8N2	H9N2
-G	-	+	-	-	-	-	0.5	0.1	0.7	0.0	0.1	0.2	0.0	0.1	0.1	0.0	0.0	0.0	0.0	0.0	0.0	0.0	2.7	0.5	0.1	0.2	0.1	0.0	2.1	0.1	0.2	0.1	0.0
Unmodified	-	-	-	-	-	-	0.2	0.1	0.0	0.0	0.1	0.1	0.0	0.0	0.0	0.0	0.1	0.0	0.0	0.0	0.0	0.0	1.2	0.5	0.2	0.0	0.0	0.0	0.5	0.0	0.1	0.1	0.1
+G	-	-	-	+	-	-	0.6	0.8	0.3	0.8	2.5	2.3	0.0	0.2	0.1	0.5	1.3	0.1	0.3	0.9	0.2	0.0	0.2	0.9	1.3	0.0	0.1	0.1	0.5	0.0	0.1	0.1	0.3
+G+S	-	-	-	+	+	-	0.1	0.1	0.1	0.2	1.5	1.5	0.2	0.7	0.0	0.1	0.3	0.0	0.2	0.7	1.1	0.1	0.2	0.2	1.4	0.0	0.1	0.1	0.6	0.0	0.1	0.1	0.1
+G+S+ivs	-	-	-	+	+	+	0.1	0.0	0.1	0.1	1.7	1.7	0.3	0.7	0.1	0.0	0.0	0.0	0.3	0.9	0.5	0.1	0.2	0.1	1.7	0.1	0.1	0.2	0.8	0.1	0.1	0.1	0.1
-G+B	-	+	+	-	-	-	0.1	0.3	1.5	0.0	0.1	0.1	0.1	0.2	0.1	0.0	0.3	0.0	0.3	0.0	0.0	0.0	0.5	0.1	0.3	0.1	0.0	0.2	3.8	0.1	0.2	1.3	0.2
+B	-	-	+	-	-	-	0.1	0.2	3.0	0.0	0.1	0.1	0.6	0.0	0.0	0.0	0.7	0.0	0.0	0.1	0.2	0.0	0.2	0.1	0.1	0.0	0.0	0.0	0.4	0.0	0.0	0.4	0.5
+B+G	-	-	+	+	-	-	0.2	0.4	9.6	0.5	1.8	2.2	3.3	0.1	0.1	0.2	1.5	0.8	0.3	0.9	0.7	0.1	0.1	0.3	1.0	0.1	0.0	0.1	0.6	0.1	0.1	0.1	0.5
+B+G+S	-	-	+	+	+	-	0.1	0.1	1.2	0.1	1.4	1.4	4.0	0.4	0.0	0.1	0.3	0.1	0.2	0.7	0.9	0.2	0.1	0.2	1.3	0.2	0.1	0.1	0.9	0.1	0.1	0.2	0.2
+B+G+S+ivs	-	-	+	+	+	+	0.1	0.1	0.2	0.1	1.4	1.5	5.2	0.4	0.1	0.1	0.1	0.0	0.3	0.8	1.1	0.1	0.2	0.1	1.4	0.3	0.1	0.2	1.0	0.2	0.2	0.2	0.1
-F-G	+	+	-	-	-	-	0.3	0.1	0.7	0.1	0.2	0.3	0.0	0.1	0.5	0.1	0.0	0.0	0.1	0.1	0.1	0.1	0.8	0.3	0.1	2.8	0.5	0.1	2.5	0.4	0.3	0.1	0.0
-F	+	-	-	-	-	-	0.1	0.0	0.0	0.1	0.1	0.1	0.0	0.1	0.2	0.1	0.1	0.0	0.1	0.0	0.0	0.0	0.4	0.2	0.1	1.4	0.4	0.1	0.6	0.5	0.2	0.1	0.0
-F+G	+	-	-	+	-	-	0.2	0.3	0.1	0.2	0.7	0.4	0.0	0.0	0.6	1.1	0.7	0.6	1.6	1.9	0.1	0.1	0.0	0.2	0.9	0.2	0.9	0.9	0.5	0.1	0.3	0.2	0.1
-F+G+S	+	-	-	+	+	-	0.1	0.0	0.6	0.1	0.3	0.3	0.2	0.2	0.1	0.1	0.1	0.2	0.9	1.2	0.5	0.4	0.1	0.1	0.6	0.3	0.3	1.8	0.7	0.1	0.1	0.1	0.0
-F+G+S+ivs	+	-	-	+	+	+	0.1	0.1	0.3	0.0	0.3	0.3	0.6	0.2	0.1	0.0	0.1	0.1	1.0	1.3	0.3	0.4	0.1	0.1	0.6	0.3	0.1	1.8	0.8	0.2	0.2	0.1	0.1
-F-G+B	+	+	+	-	-	-	0.2	0.0	0.9	0.0	0.0	0.2	0.0	0.3	0.1	0.2	0.4	0.0	0.1	0.0	0.0	0.0	0.2	0.0	0.1	0.5	0.1	0.2	4.1	0.2	0.4	0.6	0.1
-F+B	+	-	+	-	-	-	0.1	0.1	0.5	0.0	0.1	0.1	0.1	0.0	0.1	0.1	2.2	0.0	0.1	0.0	0.4	0.0	0.1	0.1	0.1	0.2	0.1	0.1	0.5	0.1	0.1	0.3	0.3
-F+B+G	+	-	+	+	-	-	0.1	0.2	2.0	0.2	0.7	0.4	0.6	0.1	0.2	0.6	6.8	0.6	1.2	1.8	1.9	0.1	0.0	0.1	0.5	0.4	0.3	0.7	0.6	0.0	0.1	0.2	0.3
-F+B+G+S	+	-	+	+	+	-	0.1	0.1	0.6	0.2	0.4	0.4	1.0	0.3	0.1	0.1	0.9	0.2	0.9	1.3	2.8	0.3	0.0	0.1	0.4	0.5	0.2	1.0	0.9	0.1	0.2	0.2	0.1
-F+B+G+S+ivs	+	-	+	+	+	+	0.1	0.1	0.2	0.1	0.4	0.4	1.3	0.4	0.1	0.0	0.2	0.1	0.9	1.4	3.7	0.3	0.1	0.0	0.4	0.6	0.1	1.1	1.0	0.2	0.2	0.2	0.1
**anti-TNP**																																	
-G	-	+	-	-	-	-	1.5	0.0	1.3	0.0	0.0	0.0	0.0	0.0	0.0	6.9	0.0	0.0	0.0	0.0	0.0	0.0	3.4	0.0	0.0	0.0	0.0	2.6	4.9	0.0	0.0	0.0	0.0
Unmodified	-	-	-	-	-	-	0.3	0.1	0.1	0.0	0.1	0.2	0.0	0.2	0.2	0.1	0.1	0.0	0.1	0.1	0.1	0.1	2.2	0.6	0.2	0.1	0.1	0.1	1.1	0.1	0.2	0.3	0.1
+G	-	-	-	+	-	-	0.4	0.7	0.3	0.7	2.6	2.6	0.0	0.3	0.0	0.4	1.2	0.0	0.3	0.9	0.2	0.0	0.3	0.9	1.3	0.0	0.0	0.1	0.8	0.0	0.1	0.1	0.2
+G+S	-	-	-	+	+	-	0.1	0.1	0.1	0.2	2.7	2.7	0.2	0.7	0.0	0.1	0.2	0.0	0.3	1.4	1.0	0.0	0.2	0.2	2.5	0.0	0.1	0.1	2.1	0.0	0.1	0.1	0.1
+G+S+ivs	-	-	-	+	+	+	0.1	0.1	0.2	0.0	2.6	2.7	0.3	0.7	0.1	0.1	0.1	0.1	0.4	1.4	0.5	0.1	0.3	0.1	2.4	0.1	0.1	0.2	1.7	0.2	0.2	0.2	0.1
-G+B	-	+	+	-	-	-	0.2	0.2	1.6	0.0	0.1	0.3	0.0	0.3	0.1	0.0	0.4	0.0	0.3	0.0	0.0	0.0	0.6	0.2	0.3	0.1	0.1	0.1	7.6	0.1	0.2	1.2	0.2
+B	-	-	+	-	-	-	0.1	0.4	1.6	0.0	0.2	0.2	0.4	0.0	0.0	0.0	0.6	0.0	0.3	0.1	0.1	0.0	0.3	0.1	0.3	0.1	0.0	0.1	1.3	0.0	0.1	0.5	0.3
+B+G	-	-	+	+	-	-	0.3	0.4	11.8	0.4	1.5	1.8	3.4	0.1	0.1	0.3	1.7	0.3	0.2	0.7	0.7	0.0	0.1	0.3	0.6	0.2	0.0	0.0	0.7	0.1	0.1	0.1	0.3
+B+G+S	-	-	+	+	+	-	0.1	0.1	1.3	0.2	1.5	1.7	4.9	0.4	0.1	0.1	0.4	0.1	0.3	1.0	1.1	0.3	0.1	0.2	1.1	0.3	0.1	0.1	1.7	0.1	0.2	0.2	0.2
+B+G+S+ivs	-	-	+	+	+	+	0.1	0.1	0.2	0.1	1.5	1.8	5.9	0.4	0.1	0.0	0.1	0.0	0.3	1.0	1.1	0.1	0.2	0.1	1.2	0.4	0.1	0.2	1.7	0.2	0.2	0.2	0.1
-F-G	+	+	-	-	-	-	0.2	0.1	0.3	0.1	0.2	0.5	0.0	0.2	0.6	0.2	0.1	0.0	0.1	0.1	0.1	0.0	0.7	0.3	0.1	2.3	0.8	0.1	5.0	0.4	0.1	0.1	0.0
-F	+	-	-	-	-	-	0.2	0.1	0.1	0.8	0.2	0.2	0.1	0.1	0.4	0.2	0.1	0.1	0.1	0.1	0.1	0.1	0.6	0.3	0.2	2.1	0.5	0.2	0.9	0.4	0.2	0.3	0.1
-F+G	+	-	-	+	-	-	0.3	0.3	0.2	0.4	1.1	0.7	0.0	0.2	0.7	1.4	0.6	0.5	1.6	2.2	0.1	0.1	0.1	0.5	1.4	0.3	1.0	0.8	1.5	0.1	0.4	0.2	0.1
-F+G+S	+	-	-	+	+	-	0.1	0.1	0.7	0.1	0.5	0.5	0.3	0.2	0.1	0.2	0.2	0.2	1.2	1.9	0.5	0.3	0.1	0.1	0.4	0.3	0.3	1.0	1.4	0.1	0.2	0.2	0.1
-F+G+S+ivs	+	-	-	+	+	+	0.0	0.0	0.3	0.0	0.5	0.5	0.7	0.2	0.0	0.0	0.0	0.0	1.2	1.9	0.3	0.4	0.1	0.0	0.5	0.3	0.1	1.1	1.3	0.2	0.1	0.2	0.0
-F-G+B	+	+	+	-	-	-	0.2	0.0	1.0	0.0	0.0	0.4	0.0	0.4	0.3	0.1	0.5	0.0	0.1	0.0	0.0	0.0	0.2	0.0	0.2	0.6	0.2	0.2	8.2	0.2	0.3	0.7	0.1
-F+B	+	-	+	-	-	-	0.2	0.2	0.4	0.0	0.1	0.1	0.1	0.1	0.2	0.2	1.4	0.0	0.2	0.1	0.2	0.0	0.1	0.1	0.2	0.4	0.2	0.2	0.9	0.1	0.2	0.3	0.2
-F+B+G	+	-	+	+	-	-	0.1	0.2	2.7	0.1	0.8	0.4	0.7	0.1	0.3	0.7	8.7	0.5	1.4	2.2	2.5	0.1	0.0	0.1	0.4	0.6	0.3	0.6	1.0	0.1	0.1	0.2	0.3
-F+B+G+S	+	-	+	+	+	-	0.1	0.1	0.5	0.2	0.4	0.4	0.9	0.3	0.1	0.1	1.0	0.2	1.0	1.6	3.0	0.3	0.0	0.1	0.3	0.5	0.2	0.9	1.6	0.1	0.2	0.2	0.1
-F+B+G+S+ivs	+	-	+	+	+	+	0.1	0.0	0.2	0.1	0.4	0.5	1.4	0.4	0.1	0.0	0.2	0.1	1.0	1.6	3.8	0.3	0.1	0.0	0.4	0.6	0.1	1.0	1.6	0.2	0.2	0.2	0.1
							Hybrid glycans																Mono- antennary glycans**						High mannose glycans				

2FF (0.4 mM 2-deoxy-2-fluoro-L-fucose), 2FG (1 mM 2-deoxy-2-fluoro-D-galactose), GNT3 (co-transfection of 1% GNT3 vector), B4GALT1/D-galactose (co-transfection of 1% B4GALT1 vector and addition of 5 mM D-galactose), ST6GALT (co-transfection of 2.5% ST6GALT vector), and in vitro sialylation (treatment of sample with recombinant ST6GALT and CMP-NANA). These resulted in significantly different derived glycosylation traits (fucosylation, bisection, galactosylation, sialylation, high-mannose, hybrid-type), which are calculated from the relative abundances of individual N-glycans. The shading of cells indicates, for each glycoform the lowest to highest abundance of glycopeptides, respectively, from light to dark.

* for complex IgG glycans, we use a nomenclature which assumes structural knowledge of the glycans based on literature. For non-complex glycans (Hybrid, Mono- antennary and High mannose), a strictly compositional nomenclature is used:

H number of hexoses (mannoses + galactoses)
N number of N-acetylhexosamines (N-acetylglucosamines + N-acetylgalactosamines)
F number of fucoses
S number of N-acetylneuraminic (sialic) acids

** for two of these glycans (N3N4 and N3H4F1), it was not possible to determine whether they were hybrid or mono-antennary.

1.2 Supplementary table 2

Acquired and in house production of human FcγRs used in SPR experiments.

Name	CD	Allotype	Source	Tag
**FcγRIa**	CD64	-	Sino Biological	HIS
**FcγRIIa**	CD32a	131 His	Sino Biological	Biotin
**FcγRIIa**	CD32a	131 Arg	Sino Biological	Biotin
**FcγRIIb**	CD32b	-	Sino Biological	Biotin
**FcγRIIIa**	CD16a	158 Phe	Sino Biological	Biotin
**FcγRIIIa**	CD16a	158 Val	Sino Biological	Biotin
**FcγRIIIb**	CD16b	NA2	Sino Biological	HIS
**FcγRIIIb**	CD16b	NA1	In-house production	Biotin
**FcγRIIIb**	CD16b	NA2	In-house production	Biotin

1.3 Supplementary Figure 1

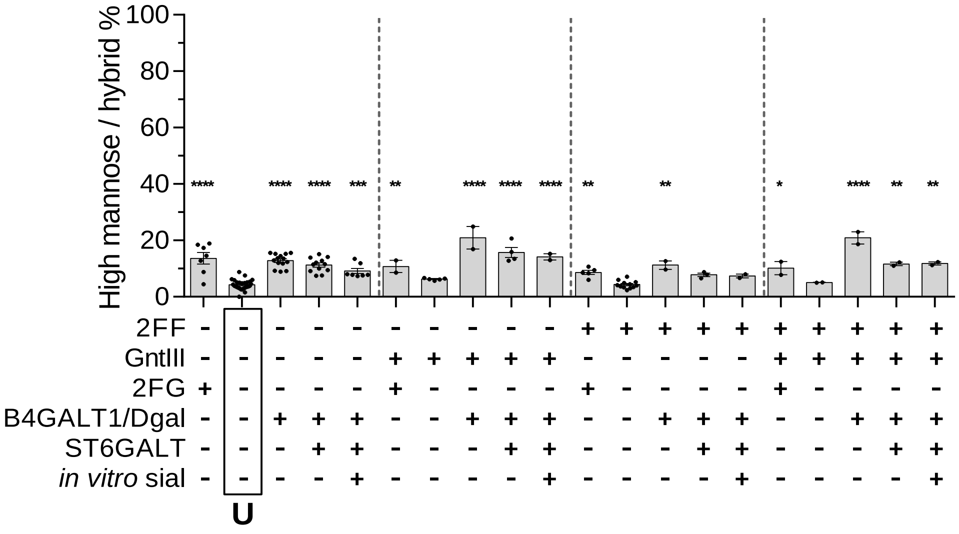

Supplementary Figure 1. Hybrid-type and high-mannose glycosylation of glycoforms
Sum of Hybrid-type and high-mannose glycopeptide abundance in the 20 different glyco-engineered IgG1 glycoforms. Data represents the mean and SEM of independent experiments; *, **, *** and **** denote a statistical significance of $p<0.05$, $p\leq0.01$, $p\leq0.001$ and $p\leq0.0001$, respectively, as tested by one-way ANOVA against unmodified IgG1, using Dunnett's multiple comparisons test.

1.4 Supplementary Figure 2

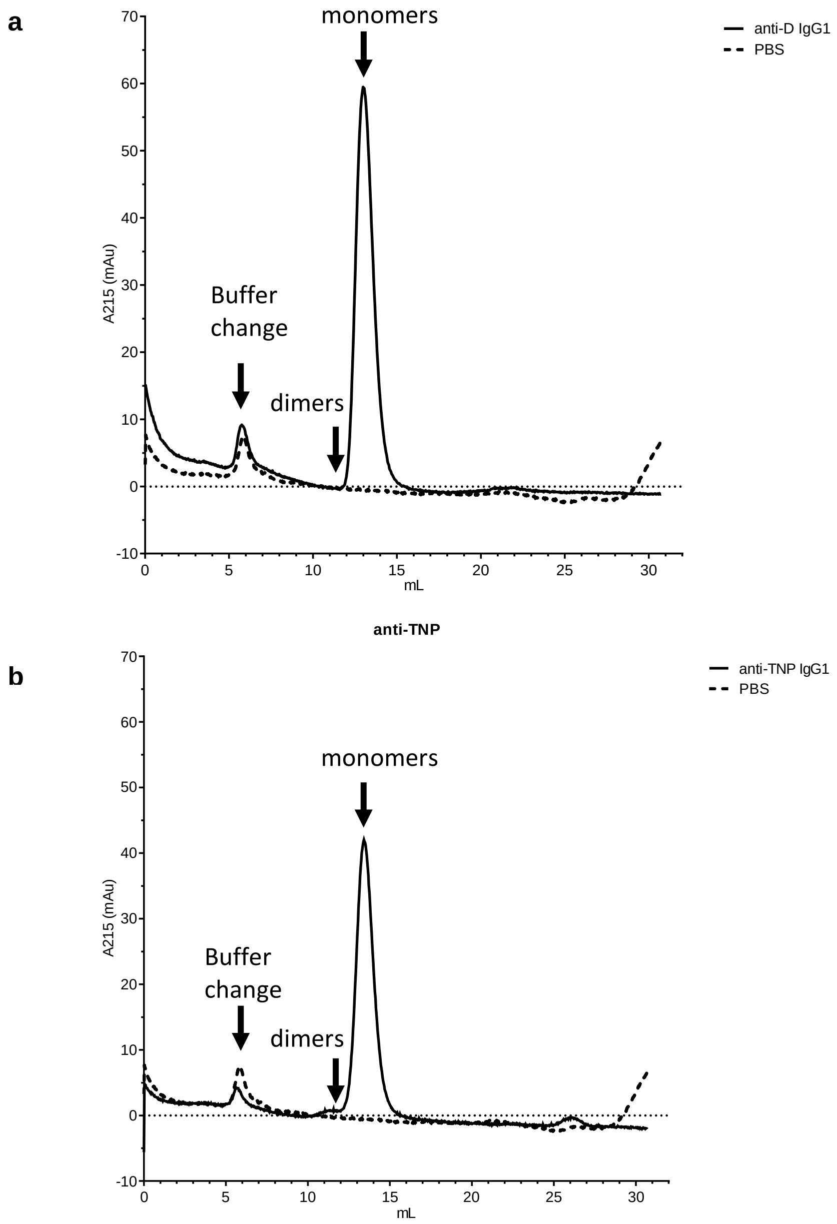

Supplementary Figure 2. HPLC analysis of purified IgG1.
Representative HP-SEC chromatograms of unmodified anti-D (a) or anti-TNP (b) IgG1 run on HPLC Superdex 200 10/300 gel filtration column, (**a**) shows for anti-D only a monomeric peak is observed and no dimeric peak, (**b**) shows for anti-TNP besides the large monomeric peak a small dimeric peak (<2% of total).

1.5 Supplementary Figure 3

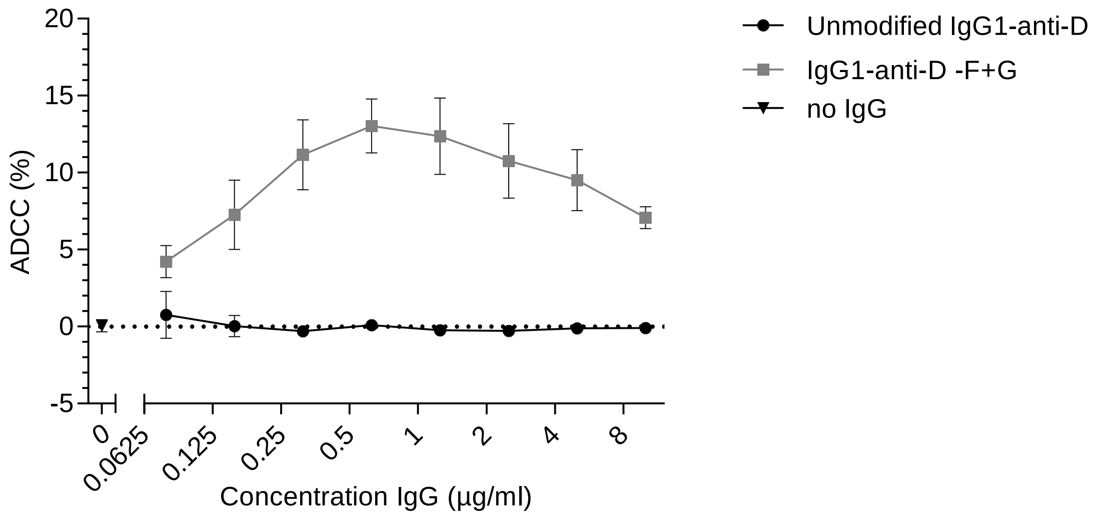

Supplementary Figure 3. Titration ADCC
Titration of IgG glycoforms in ADCC assay; data represents means and SD of two donors, (-F+G: hypofucosylated and hypergalactosylated IgG1.

1.6 Supplementary Figure 4

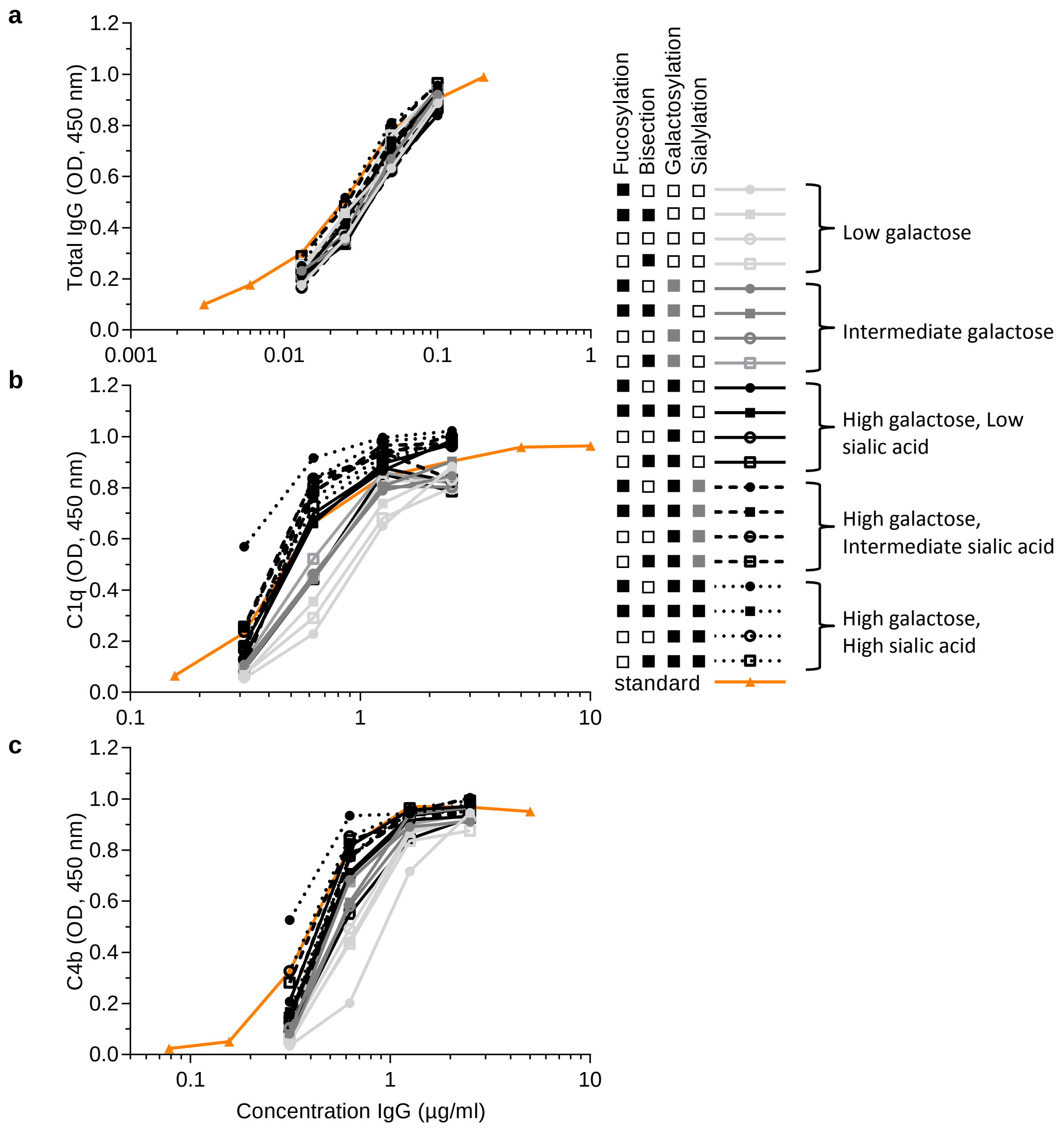

Supplementary Figure 4. IgG, C1q binding and C4b deposition as measured by ELISA
Raw data and control ELISA data of complement ELISA with HSA-TNP coat, followed by incubation with aTNP IgG1 glycoforms and subsequent complement deposition by incubation with human. **a**) anti-total human IgG, **b**) anti-C1q and **c**) anti-C4b detection respectively. Data are representative of n=6. As legend describes: the line color from light grey to black represents low to high galactosylation respectively, solid line to more dashed represents low to high sialylation respectively, open or filled symbols represent low or high fucose respectively and circle or square symbols represent low or high bisection respectively. From panels **b** and **c** we can conclude that all high galactosylated and sialylated are more active than low galactosylated and sialylated which are relatively more left or right of the standard curve respectively.

1.7 Supplementary Figure 5

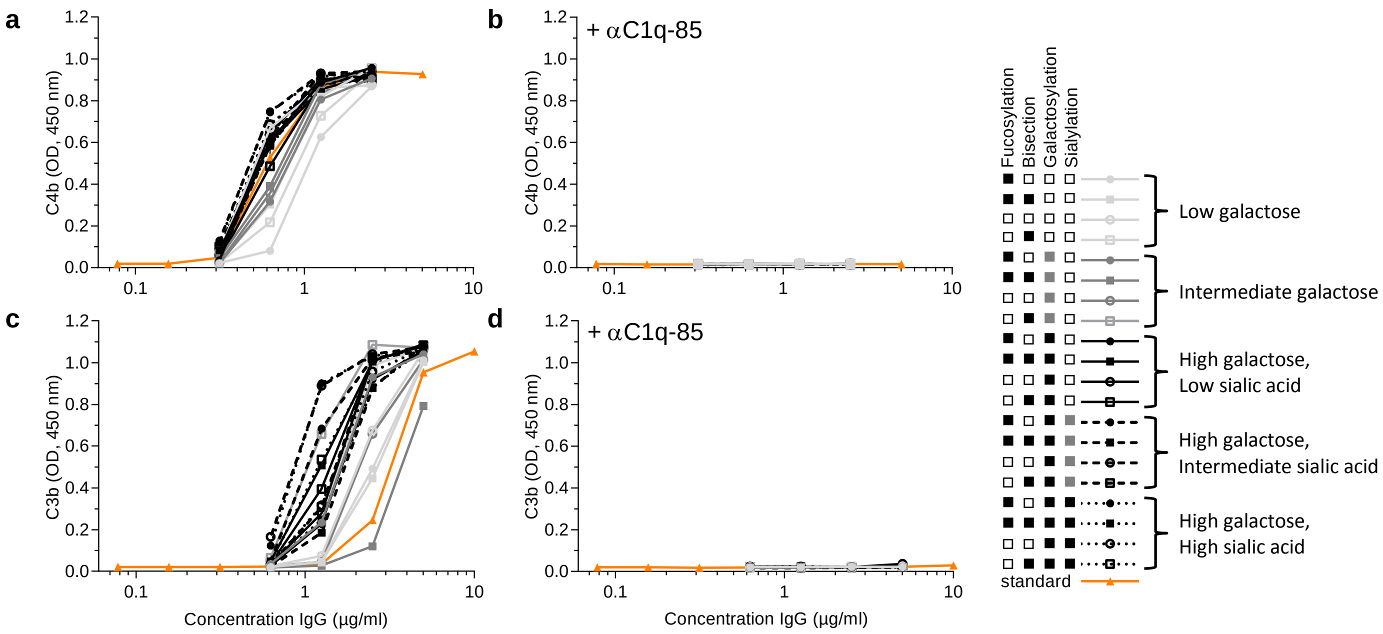

Supplementary Figure 5. C4b and C3b deposition requires C1q activity
Complement ELISA with HSA-TNP coat, incubation with aTNP IgG1 glycoforms, subsequently incubation with pooled human serum, HRP-labeled-anti-C4b (**a, b**) or biotin-anti-C3b and subsequent strep-HRP (**c, d**), (**a, c**) control plates (**c, d**) incubated with anti-C1q-85 blocking antibody, data are representative of n = 2. As legend describes: the line color from light grey to black represents low to high galactosylation respectively, solid line to more dashed represents low to high sialylation respectively, open or filled symbols represent low or high fucose respectively and circle or square symbols represent low or high bisection respectively. No C4b or C3b deposition was detected with C1q blocking, both can respectively be found when the lectin or alternative pathway is activated, showing no involvement of either pathway.

1.8 Supplementary Figure 6

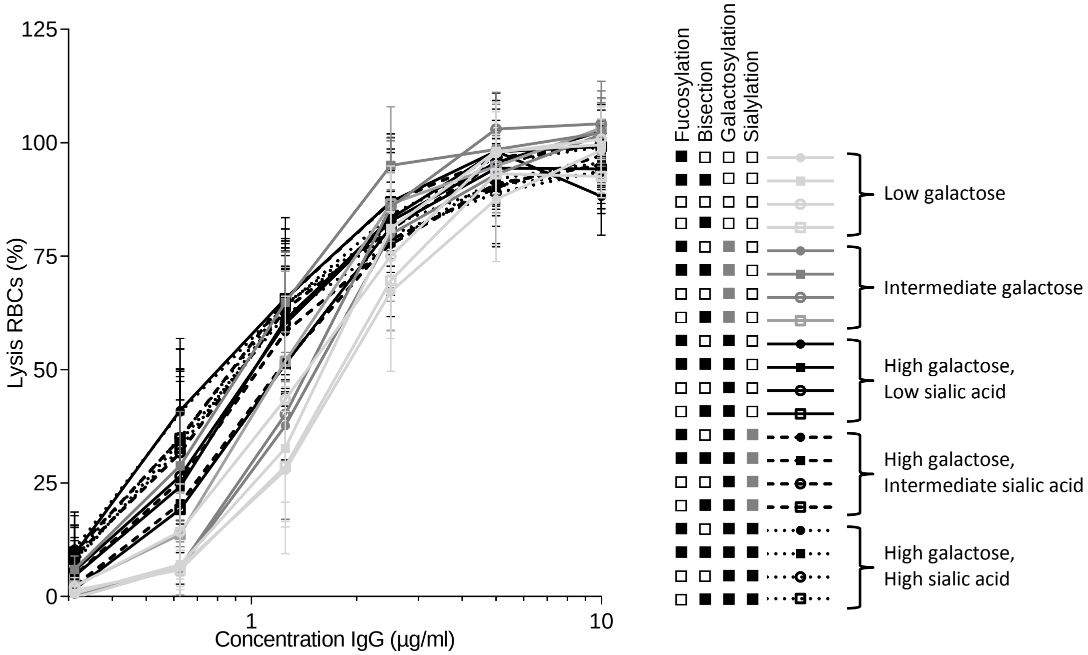

Supplementary Figure 6. TNP labelled RBC incubated with anti-TNP IgG glycoforms and pooled human serum. Lysis was measured as hemoglobin release into the supernatant by spectrophotometer (OD450 - OD650) and calculated relative to signal of maximum lysis control (2.5% saponine). Data are means and SD of 2 representative experiments 3 in total, each carried out in duplo. As legend describes: the line color from light grey to black represents low to high galactosylation respectively, solid line to more dashed represents low to high sialylation respectively, open or filled symbols represent low or high fucose respectively and circle or square symbols represent low or high bisection respectively. We can conclude that all high galactosylated and sialylated IgG1 are more active than low galactosylated and sialylated IgG1 which are relatively more left or right respectively.

1.9 Supplementary Figure 7

a

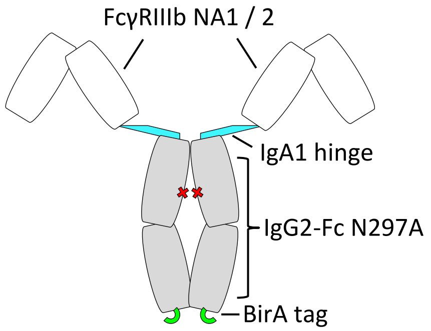

b

```
Fc-FcgRIIIb_NA1   MWQLLLPTAL LLLVSAGMRT EDLPKAVVFL EPQWYRVLEK DSVTLKCQGA YSPEDNSTQW  60
Fc-FcgRIIIb_NA2   MWQLLLPTAL LLLVSAGMRT EDLPKAVVFL EPQWYSVLEK DSVTLKCQGA YSPEDNSTQW  60

Fc-FcgRIIIb_NA1   FHNENLISSQ ASSYFIDAAT VDDSGEYRCQ TNLSTLSDPV QLEVHVGWLL LQAPRWVFKE  120
Fc-FcgRIIIb_NA2   FHNESLISSQ ASSYFIDAAT VNDSGEYRCQ TNLSTLSDPV QLEVHIGWLL LQAPRWVFKE  120

Fc-FcgRIIIb_NA1   EDPIHLRCHS WKNTALHKVT YLQNGKDRKY FHHNSDFHIP KATLKDSGSY FCRGLVGSKN  180
Fc-FcgRIIIb_NA2   EDPIHLRCHS WKNTALHKVT YLQNGKDRKY FHHNSDFHIP KATLKDSGSY FCRGLVGSKN  180

Fc-FcgRIIIb_NA1   VSSETVNITI TQEFPVPSTP PTPSPSTPPT PSPSCCHAPP VAGPSVFLFP PKPKDTLMIS  240
Fc-FcgRIIIb_NA2   VSSETVNITI TQEFPVPSTP PTPSPSTPPT PSPSCCHAPP VAGPSVFLFP PKPKDTLMIS  240

Fc-FcgRIIIb_NA1   RTPEVTCVVV DVSHEDPEVQ FNWYVDGVEV HNAKTKPREE QFASTFRVVS VLTVVHQDWL  300
Fc-FcgRIIIb_NA2   RTPEVTCVVV DVSHEDPEVQ FNWYVDGVEV HNAKTKPREE QFASTFRVVS VLTVVHQDWL  300

Fc-FcgRIIIb_NA1   NGKEYKCKVS NKGLPAPIEK TISKTKGQPR EPQVYTLPPS REEMTKNQVS LTCLVKGFYP  360
Fc-FcgRIIIb_NA2   NGKEYKCKVS NKGLPAPIEK TISKTKGQPR EPQVYTLPPS REEMTKNQVS LTCLVKGFYP  360

Fc-FcgRIIIb_NA1   SDIAVEWESN GQPENNYKTT PPMLDSDGSF FLYSKLTVDK SRWQQGNVFS CSVMHEALHN  420
Fc-FcgRIIIb_NA2   SDIAVEWESN GQPENNYKTT PPMLDSDGSF FLYSKLTVDK SRWQQGNVFS CSVMHEALHN  420

Fc-FcgRIIIb_NA1   HYTQKSLSLS PGKGLNDIFE AQKIEW                                    446
Fc-FcgRIIIb_NA2   HYTQKSLSLS PGKGLNDIFE AQKIEW                                    446
```

c

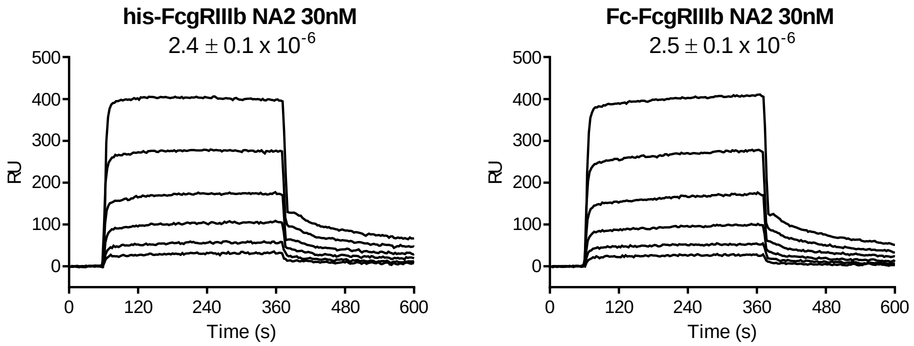

Supplementary Figure 7. FcγRIIIb-Fc Fusion construct
Model (**a**) and amino acid sequence (**b**) of the FcγRIIIb_NA1_IgA1Hinge_IgG2-Fc_N297A_BirA-tag and FcγRIIIb_NA2_IgA1Hinge_IgG2-Fc_N297A_BirA-tag, shown in white; FcγR, yellow; NA1/NA2 differences, blue; IgA1 hinge, grey; IgG2Fc, red; N297A substitution, green; BirA-tag, **c, d**). SPR analysis on IBIS MX96 of binding of unmodified IgG to the his-tag captured FcγRIIIb NA2 (**c**) or in house produced FcγRIIIb_NA2-Fc-fusion (**d**) shows comparable binding affinities in K_D and SD (n = 3).
